# Topical herbal medicine as treatment for chemotherapy-induced peripheral neuropathy: a systematic review of randomized controlled trials

**DOI:** 10.3389/fonc.2026.1920042

**Published:** 2026-09-28

**Authors:** Carrie Shao, Cassie Shao, Andrew Haggarty, Mingxiao Yang, Ting Bao

**Affiliations:** 1 University of Massachusetts Chan Medical School, Worcester, MA, United States; 2 Lamar Soutter Library, University of Massachusetts Medical School, Worcester, MA, United States; 3 School of Biological Sciences, College of Science, Nanyang Technological University, Singapore, Singapore; 4 Departments of Supportive Care Medicine and Medical Oncology, City of Hope Comprehensive Cancer Center, Duarte, CA, United States

**Keywords:** cancer survivorship, chemotherapy-induced peripheral neuropathy, herbal medicine, integrative oncology, randomized controlled trials, symptom management, topical therapy

## Abstract

**Introduction:**

Chemotherapy-induced peripheral neuropathy (CIPN) is a common neurological complication of cancer treatment that worsens function and quality of life. Topical herbal medicines are increasingly used as localized interventions for neuropathic symptoms; however, their clinical efficacy remains uncertain. This systematic review evaluated the effectiveness of topical herbal formulations for CIPN in cancer populations.

**Methods:**

We systematically searched Scopus, MEDLINE (Ovid), CINAHL, and Cochrane CENTRAL for randomized controlled trials assessing the use of topical herbal formulations in adults with CIPN. Outcomes included validated measures of CIPN pain, overall severity, sensory function, and adverse events. Risk of bias was assessed using the Cochrane Risk of Bias tool implemented in RevMan 5.3, and all procedures were conducted in accordance with PRISMA guidelines.

**Results:**

Five trials involving 201 participants were included, with an overall moderate risk of bias due to limited blinding and underpowered sample sizes. In breast cancer patients, henna vs. standard care significantly improved the overall severity of oxaliplatin-induced peripheral neuropathy, as measured by the CIPN Assessment Tool (*p* < 0.001), with significant reductions in sensory and functional subdomain scores. In a mixed-cancer population, *Costus* sp. oil vs. placebo significantly reduced neuropathic pain intensity, as measured by the Visual Analog Scale (VAS) (*p* = 0.001), and improved Functional Assessment of Cancer Therapy/Gynecologic Oncology Group - Neurotoxicity (FACT/GOG-Ntx) scores (*p* = 0.028). Trials evaluating ajwain cream, *Citrullus colocynthis* oil, and a multi-compound essential oil formulation did not demonstrate statistically significant improvements in CIPN severity, pain, or quality of life compared with placebo. None of the included studies incorporated quantitative sensory or functional testing. All interventions were well tolerated, with only mild localized skin reactions reported.

**Discussion:**

Despite modest benefits from certain herbal treatments in reducing CIPN pain and sensory symptoms, current evidence is limited by small sample sizes, methodological heterogeneity, and the lack of objective outcome measures. Rigorous, adequately powered RCTs with objective CIPN measurements are needed.

## Introduction

Chemotherapy-induced peripheral neuropathy (CIPN) is a common and dose-limiting side effect that affects up to 60% of people with cancer receiving neurotoxic chemotherapeutic agents (1). However, the incidence, severity, and symptom profile of CIPN vary considerably depending on the causative agent. For example, oxaliplatin characteristically produces an acute neuropathy marked by cold allodynia, often within hours of infusion (6). Despite this heterogeneity, CIPN most commonly presents with sensory symptoms, including pain, numbness, and paresthesias, with motor deficits occurring less frequently, and can substantially impair quality of life, often necessitating dose reduction or discontinuation of chemotherapy (2, 3, 6). However, effective management options remain limited. To date, duloxetine is the only treatment recommended by the American Society of Clinical Oncology (ASCO) for post-treatment CIPN pain relief. Its benefits are modest and accompanied by adverse effects, including fatigue, drowsiness, and sleep disturbances (4). A growing body of research also highlights the promise of nonpharmacologic interventions, i.e., exercise, acupuncture, and yoga, though definitive clinical evidence remains emerging (5). Consequently, additional management strategies for CIPN are needed across cancer care settings.

Recently, interest in complementary and integrative medicine approaches as potential treatments for CIPN has increased. Certain botanical products have demonstrated neuroprotective, anti-inflammatory, antioxidant, and axonal-supportive properties relevant to the pathophysiology of CIPN (6–9). Given safety concerns about herb-drug interactions, topical application of herbal ingredients represents a viable and acceptable option for this patient population, as it bypasses oral absorption, reduces systemic exposure, and thereby lowers the risk of adverse drug reactions. Despite emerging clinical evaluations, the efficacy and safety of topical herbal treatments for CIPN remain unclear. A rigorous appraisal of the current evidence is therefore needed to provide translational insights that can inform clinical practice and guide future high-quality research.

To identify and synthesize relevant evidence, we conducted a systematic review of randomized clinical trials evaluating topical herbal interventions administered exclusively as direct-application formulations for the treatment of CIPN. Specifically, we assessed the methodological quality of available studies, summarized the herbal ingredients and application protocols used, evaluated their efficacy, and identified gaps in the literature to inform future research. By concentrating solely on single modality topical herbal therapy, this review seeks to determine whether this approach offers meaningful benefits for patients suffering from CIPN.

## Methods

The population, intervention, comparator, and outcome (PICO) framework was used to guide the development of the eligibility criteria for this systematic review. The review was reported in accordance with the preferred reporting items for systematic reviews and meta-analyses (PRISMA) guidelines.

### Search strategy

A literature search was conducted in four databases: Scopus (Elsevier), MEDLINE (Ovid), CINAHL (EBSCO), and the Cochrane Central Register of Controlled Trials, from inception to August 2025, with an updated search in April 2026. Because terminology describing complementary therapies varies considerably across the literature, we developed a search strategy that combined herbal medicine terms and their synonyms adapted from the comprehensive complementary, alternative, and integrative medicine (CAIM) search string developed by Ng et al. with terms related to CIPN and topical administration to maximize retrieval of relevant studies (10). Detailed search strategies for each database are provided in **Supplementary File 1**. Search results from all databases were imported into Covidence, where duplicate records were identified and removed using the platform’s automated duplicate detection function prior to title and abstract screening.

### Selection criteria

Following duplicate removal, two reviewers (CS and CS) independently screened titles and abstracts of all retrieved records in Covidence. Studies considered potentially eligible underwent independent full-text review by the same reviewers. Any disagreements regarding study eligibility were resolved through discussion until consensus was reached. Studies that met the following criteria were included: 1) randomized controlled trials published in English; 2) adult cancer patients or survivors who are undergoing or have undergone chemotherapy, and are diagnosed with CIPN; 3) herbal medicine of any geographic or ethnic origin intended for external application; 4) a valid control group, such as placebo or standard care; 5) assessed one or more CIPN-related outcomes including overall severity, neuropathic pain, quality of life, sensory or functional studies using validated instruments. Interventions were considered herbal medicine if they contained, as an active ingredient, whole plants, parts of plants, or plant-derived preparations. Preparations consisting of single isolated compounds (e.g., menthol, capsaicin, cannabidiol) were excluded. Trials that employed a crossover design or combined topical herbal medicine with other therapeutic modalities, such as heat therapy in footbaths, were excluded.

### Data extraction

Two reviewers (CS and CS) independently extracted data using a prespecified data collection form designed in accordance with the Methodological Expectations of Cochrane Intervention Reviews (11). Key data items included study metadata, patient characteristics, cancer and chemotherapy types, topical herbal formulations and their dosages, control groups, outcome metrics, and notable findings. Any discrepancies in data collection were resolved through discussion. We transcribed the study information from the data collection form into Review Manager (RevMan) software (version 5.4) to generate a table of study characteristics (12).

### Data analysis

We conducted risk-of-bias assessments using the Cochrane Risk of Bias tool for randomized controlled trials, as implemented in RevMan software version 5.4 (Cochrane), to appraise each study’s randomization process, allocation concealment, blinding of participants and study personnel, blinding of outcome assessment, reporting of outcomes, missing outcome data, and other sources of bias. Each category was assigned a rating of low, unclear, or high risk of bias based on information gathered from the study protocol and final report. The results of the risk-of-bias assessment were visualized using traffic light graphs generated in RevMan.

A meta-analysis was not performed due to the limited number of included studies and the considerable variation in outcome measures and intervention composition. Owing to this heterogeneity, we conducted a narrative synthesis of the included studies. Outcome measures unrelated to those of our interest (i.e., CIPN severity, neuropathic pain, sensory function) were recorded but not synthesized.

## Results

### Characteristics of included studies

The search strategy yielded 751 results. After removal of duplicates, 358 remained for title and abstract screening. After initial eligibility screening, full texts of six studies were retrieved, of which one did not meet our inclusion criteria, leaving five RCTs eligible for inclusion in this systematic review. The study flow is illustrated in **Figure 1**.

**Figure 1:**
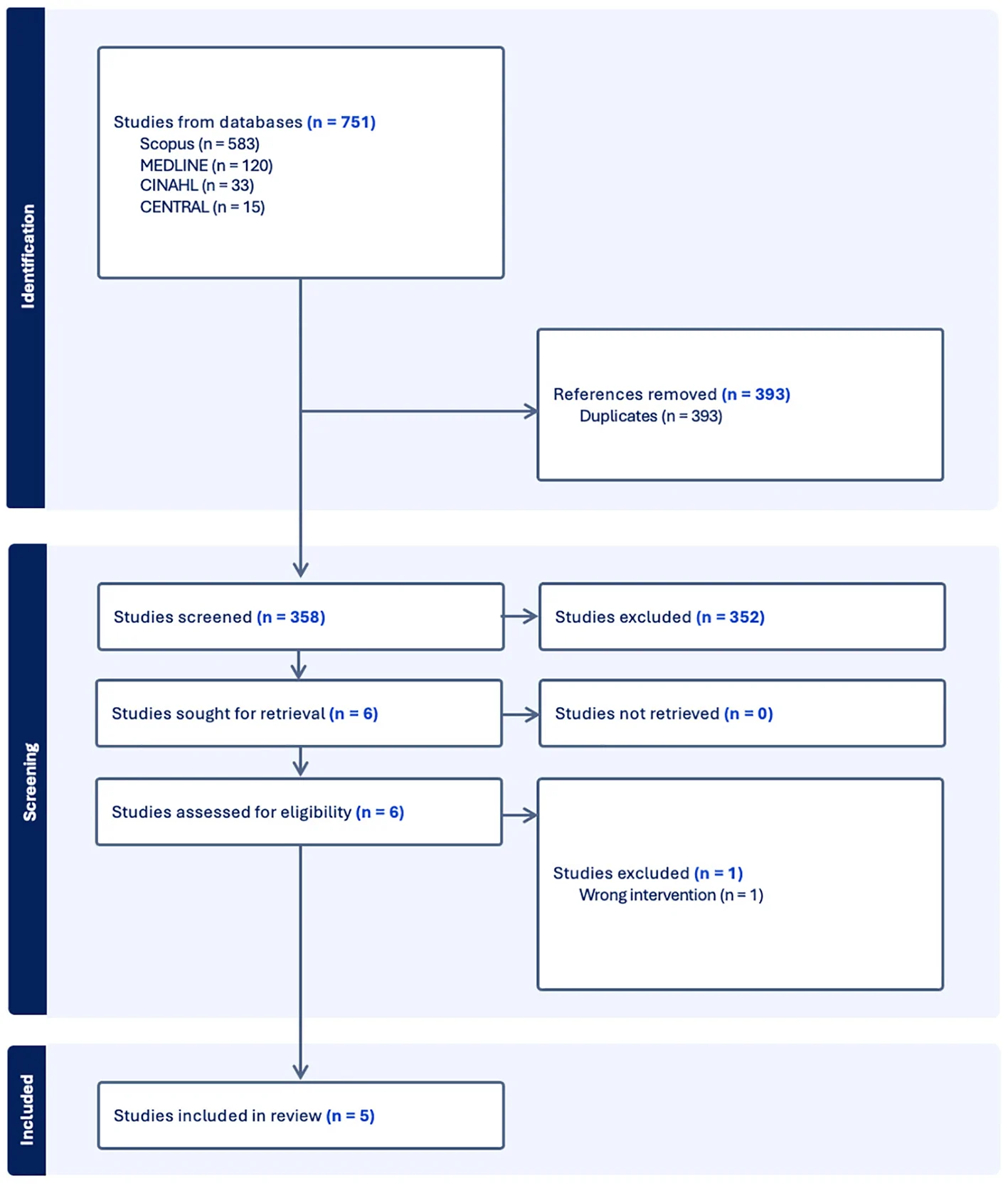
PRISMA diagram.

**Table 1** details the characteristics of the five included RCTs, published between 2019 and 2024. These studies encompass sample sizes ranging from 27 to 60 participants, with a cumulative total of 201 individuals living with cancer or cancer survivors from Iran, Turkey, or the USA (13–17).a Among these, three studies involved patients with breast cancer (n = 73), two with gastrointestinal cancers (n = 69), one with lung cancer (n = 6), one with prostate cancer (n = 3), and one with unspecified cancer type data (n = 50). Participants were undergoing or had received oxaliplatin-based chemotherapy in two trials (n = 64), taxane-based therapy in two trials (n = 60), or unspecified regimens in two trials (n = 77). Topical herbal interventions included henna powder (13), ajwain cream (14), Costus sp. oil (15), essential oil (16), or C. colocynthis fruit extract oil (17), administered over 4 weeks (14, 15, 17), 6 weeks (16), or twice across two chemotherapy cycles (13).

**Table 1:** Characteristics of included studies.

Author, year	Participants	Intervention	Comparator	Outcome assessment (Timepoint)	Key findings	Adverse events
Arslan, 2020 (13)	Female cancer patients with grade II or III neurotoxicity on CTCAE receiving oxaliplatin-based chemotherapy (n=60)	50g Pure henna powder applied after the 2^nd^ & 3^rd^ 15-day chemotherapy cycle	Standard care	CIPNAT (after 2^nd^, 3^rd^, and 4^th^ chemotherapy cycle)	Reduction in CIPNAT vs. control (p < 0.001)	None
Heydarirad, 2024 (14)	Cancer patients diagnosed clinically with CIPN receiving or w/history of taxane chemotherapy within past 2 months (n=30)	5g Ajwain cream twice daily x 4 weeks	Placebo: paraffin oil cream, with tubes washed w/ajwain distillate to simulate aroma	CIPNAT (0, 4 weeks)	No significant difference vs. placebo (p=0.372)	Mild erythema and pruritus
Heydarirad, 2020 (15)	Cancer patients with clinically diagnosed CIPN undergoing chemotherapy or w/history of undergoing chemotherapy within past 6 months (n=50)	*Costus* sp. oil ointment three times daily x 4 weeks	Placebo: liquid & solid paraffin w/green dye & sesame oil to mimic smell	VAS for neuropathic pain; FACT/GOG-Ntx; PNQ; feasibility (0, 4 weeks)	Reduction VAS vs. placebo (p=0.001) Reduction in FACT/GOG-Ntx vs. placebo (p=0.028)	Mild erythema and pruritus
Langley-Brady, 2023 (16)	Breast cancer survivors 3 months post chemotherapy with self-reported CIPN and a mean score ≥1 on SF-MPQ-2 (n=27)	5ml Topical essential oil blend in *Simmondsia chinensis* (Jojoba) three times daily x 6 weeks	Placebo: Jojoba alone	VAS for pain (daily) SF-MPQ-2 (0, 1, 2, 3, 4, 5, 6 weeks) EORTC QLQ-CIPN20; QLACS (0, 3, 6 weeks)	No significant differences vs. placebo	Assessed but not reported
Rostami, 2019 (17)	Breast cancer patients with diagnosed CIPN and received chemotherapy within past 2 months (n=34)	2ml Topical formulation of C. *colocynthis* fruit extract oil boiled in sesame oil twice daily x 4 weeks	Placebo: sesame oil alone	FACT/GOG-Ntx (0, 4 weeks)	No significant difference vs. placebo	None

The primary outcomes examined across studies included 1) CIPN overall symptom severity, assessed using the Chemotherapy-Induced Peripheral Neuropathy Assessment Tool (CIPNAT) or the Functional Assessment of Cancer Therapy/Gynecologic Oncology Group-Neurotoxicity scale (FACT/GOG-Ntx) (13, 14, 17), 2) CIPN pain, assessed with the visual analog scale (VAS) and the Short-Form McGill Pain Questionnaire-2 (SF-MPQ-2) (16), and 3) quality of life using the Quality of Life Chemotherapy-Induced Peripheral Neuropathy-20 Questionnaire (EORTC QLQ-CIPN20) and the Quality of Life Adult Cancer Survivor Questionnaire (QLACS) (16). One feasibility study primarily evaluated treatment acceptability and compliance, but included VAS, FACT/GOG-Ntx, and the Patient Neurotoxicity Questionnaire (PNQ) as exploratory outcomes (15). No studies included sensory laboratory tests or functional assessments of balance, gait and mobility. Four studies reported adverse events (13–15, 17).

### Risk of bias assessment

**Figure 2** demonstrates the methodological quality of the studies included. All trials exhibited a low risk of bias for random sequence generation and allocation concealment. In one study, participants were not blinded because the intervention was compared with standard care rather than a placebo, resulting in a high risk of performance bias (13). One additional study was judged to have an unclear risk of performance bias because the extent of personnel blinding was insufficiently described (16). Blinding of outcome assessment was judged as unclear risk in 2 of 5 studies (13, 16). In one study, the primary outcome was patient-reported and the potential for biased outcome assessment could not be excluded (13), whereas the procedures for blinding outcome assessment were incompletely described in the other (16). Incomplete outcome data and selective reporting were generally considered low risk. Other potential sources of bias were unclear in several studies, primarily due to pilot study designs that were not powered for efficacy or limited information regarding intervention standardization.

**Figure 2:**
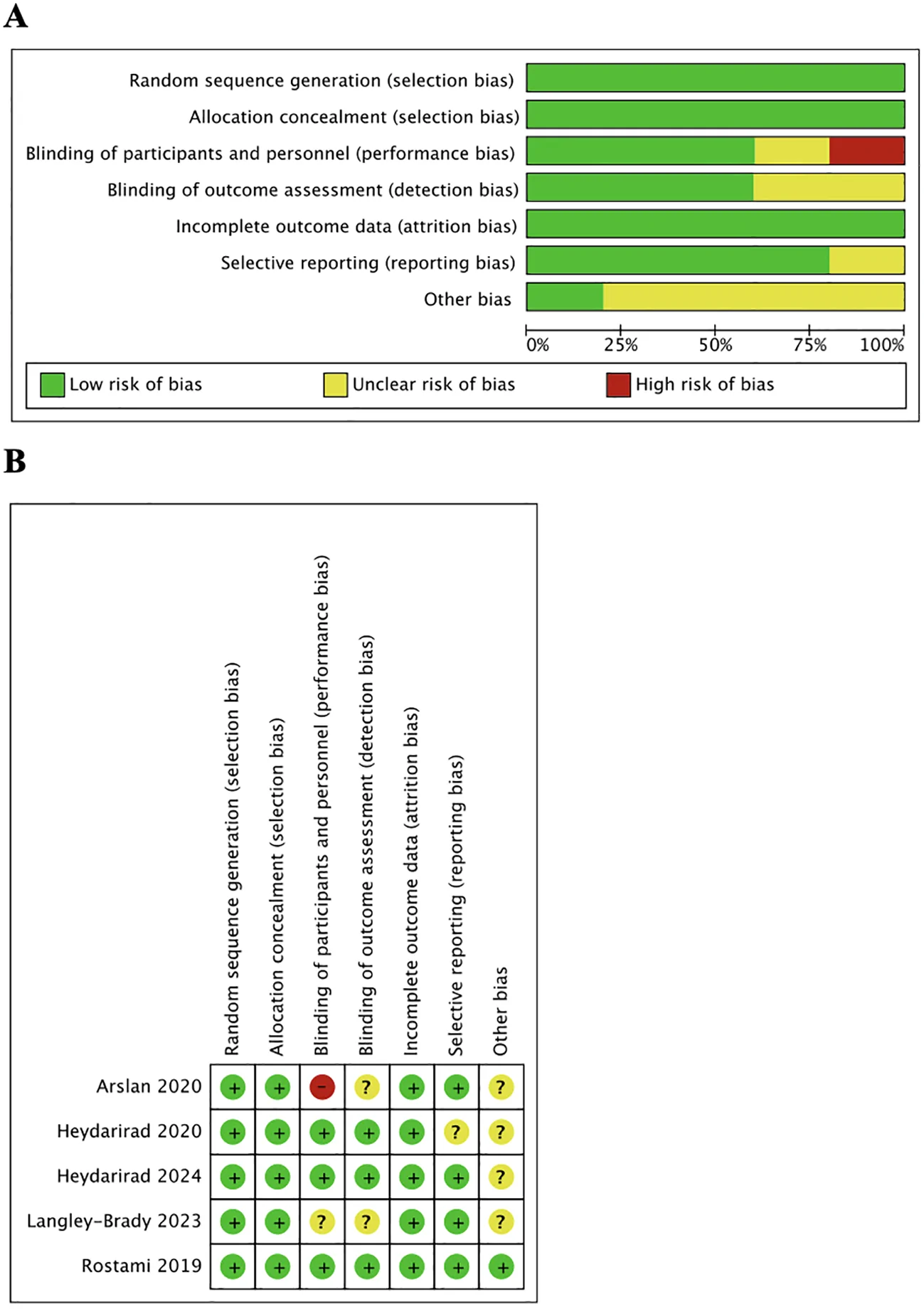
Risk of bias assessment: **(A)** Risk of bias graph; **(B)** Risk of bias summary.

### Effects of interventions by outcome

#### CIPN overall severity

Four trials assessed CIPN overall severity using validated patient-reported outcome measures (13–15, 17), including the CIPNAT and the FACT/GOG-Ntx scale. Two trials reported statistically significant improvements in CIPN severity following topical herbal interventions compared with control conditions (13, 15), whereas two others found no significant between-group differences (14, 17).

In a pilot trial involving women receiving oxaliplatin-based chemotherapy (n = 60), topical henna applied following two consecutive chemotherapy cycles, compared with standard care, resulted in a statistically significant group x time interaction for total CIPNAT scores favoring the intervention (F = 10.78, *p* < 0.001; η^2^ = 0.169) (13). In contrast, a separate pilot trial in patients with taxane-induced peripheral neuropathy (n = 30) found no statistically significant between-group difference in change in CIPNAT scores following four weeks of twice-daily application of ajwain cream compared with placebo (mean difference = 0.83; *p* = 0.372) (14).

Among trials utilizing the FACT/GOG-Ntx scale, one study of breast cancer patients with oxaliplatin or taxane-induced peripheral neuropathy found no improvement in total FACT/GOG-Ntx scores following four weeks of twice-daily topical *Citrullus colocynthis* oil vs. placebo (mean change (SD): 1.05 (1.36) vs. 2.40 (1.90); *p* = 0.879) (17). In a feasibility trial of similar duration, topical *Costus* sp. oil applied three times daily for four weeks was associated with a statistically significant reduction in total FACT/GOG-Ntx scores compared with placebo (mean: 18.52 vs. 12.87; *p* = 0.028) in cancer patients with CIPN; however, this finding emerged from exploratory analyses in a study not powered for efficacy (15).

#### CIPN pain

Two trials assessed CIPN-related pain using the VAS (15, 16), with one trial additionally assessing pain characteristics using the SF-MPQ-2 (16). One study reported a significant reduction in neuropathic pain following a topical herbal intervention compared with controls (15), whereas the other found no such difference (16).

As part of the exploratory analyses of the placebo-controlled feasibility trial, four weeks of topical *Costus* sp. oil vs. control led to a statistically significant reduction in VAS pain scores (mean: 4.80 vs. 5.73; *p* = 0.001) (15). In contrast, among breast cancer survivors with CIPN three months post-chemotherapy, six weeks of topical essential oil three times daily did not result in statistically significant between-group differences in change in VAS or SF-MPQ-2 pain scores over time (*p* = 0.196 and *p* = 0.480, respectively), despite a decrease in VAS scores observed within the intervention group (16).

#### Quality of life

Quality of life was assessed in one trial using both a CIPN-specific and a global survivorship quality of life measure (16). As secondary outcomes of the topical essential oil trial, no statistically significant improvement in CIPN-specific quality of life was observed compared with placebo among breast cancer survivors, as assessed by the EORTC QLQ-CIPN20 (*p* = 0.885). Similarly, global quality of life assessed using the QLACS instrument showed no statistically significant between-group differences in total or subdomain scores, including pain, energy level, and negative feelings (*p* > 0.05 across domains).

#### Sensory and motor function

No included trials assessed sensory function using objective laboratory tests for CIPN, such as nerve conduction studies or quantitative sensory testing, nor did any trials employ biobehavioral assessments to evaluate functional impairments in mobility, balance, or gait. However, several studies reported sensory, motor, and functional subdomain scores within broader patient-reported measures of CIPN (13, 15, 17).

In the topical henna study, analyses of CIPNAT subdomains demonstrated a significant improvement in sensory symptoms over time in the intervention group compared with standard care (F = 10.86, *p* <.001; η^2^ = 0.17) (13). Functional subscores, including fine motor and general daily activity, also showed significant improvements in the henna group relative to controls (*p* = 0.001 and *p* < 0.001, respectively). Conversely, motor symptom scores did not demonstrate a statistically significant group x time interaction (F = 0.912, *p* = 0.344; η^2^ = 0.016). In the study evaluating topical *C. colocynthis* oil, sensory, motor, and functional domains of the FACT/GOG-Ntx scale showed no statistically significant between-group differences following the intervention, with no significant improvements observed in either the intervention or placebo groups (*p* > 0.05 across domains) (17). Similarly, in the exploratory analyses of the *Costus* sp. oil feasibility trial, no significant differences were observed in the sensory and motor dimensions of PNQ compared with placebo (*p* = 0.293 and *p* = 0.495, respectively) (15).

### Adverse events

All included trials monitored adverse events throughout the intervention periods; however, one study did not report adverse event outcomes (16). Among trials that reported safety data, two studies noted isolated cases of mild erythema and pruritus at the application sites (14, 15), while two studies found no adverse events entirely (13, 17). In the Ajwain cream trial, four cases of redness and one case of itching were reported in the intervention group compared with one and two cases, respectively, in the placebo group (14). In the *Costus* sp. oil trial, one case each of erythema and pruritus was reported in the intervention group, with no adverse events reported in the placebo group (15). No serious adverse events, including systemic effects, clinically significant laboratory abnormalities, or hospitalizations attributable to the topical herbal interventions were documented across trials.

## Discussion

Our systematic review synthesized evidence from RCTs evaluating topical herbal medicines as single-modality interventions for CIPN. Across five trials involving 201 participants from Iran, Turkey, and the United States, the evidence for benefit was heterogeneous and remains limited in both scale and methodological consistency. Within this small body of literature, topical henna significantly reduced overall CIPN severity in women with oxaliplatin-induced peripheral neuropathy, while *Costus* species oil was associated with both reduced neuropathic pain intensity and overall CIPN severity, although this evidence was derived from a feasibility trial not powered to assess efficacy. In contrast, ajwain cream, *C. colocynthis* oil, and a multi-essential oil formulation did not demonstrate statistically significant improvements in CIPN-related or QoL outcomes compared with placebo. These findings suggest that only specific herbal treatments may lead to symptomatic relief in specific contexts. Definitive conclusions are constrained by the size and quality of the evidence base. This is consistent with broader literature evaluating complementary approaches for CIPN, where promising preliminary findings often require confirmation in larger, more rigorous trials before clinical recommendations can be made (9, 18, 19).

One of the most consistent findings across the included trials was the favorable safety profile of topical herbal medicines. Reported adverse events were minimal and limited to mild localized skin reactions. Given that duloxetine, the only pharmacologic agent currently recommended for CIPN, can produce systemic side effects of fatigue, nausea and insomnia (4), topical herbal therapies may therefore represent a low-risk adjunctive strategy for symptomatic management of CIPN, particularly when systemic treatments are poorly tolerated. Additionally, because CIPN symptoms often persist long after chemotherapy completion, low-risk interventions that can be self-administered at home may offer long term practical advantages.

The influence of placebo effect is an important consideration when interpreting the findings. Neuropathic pain is highly susceptible to modulation by expectancy responses, and topical therapies may enhance these responses through contextual factors surrounding treatment delivery such as tactile stimulation, sensory cues like odor, or even the ritual of repeated application (20–22). In the essential oil trial, improvements in CIPN pain occurred in both intervention and placebo groups without statistically significant between-group differences. Given the six-week study duration, some improvement may have reflected the natural regression of the disease; however, a placebo response is an equally plausible explanation. These findings underscore the importance of carefully designed placebo controls in distinguishing the specific effects of topical interventions on pain-related outcomes, while also bringing to light the clinical relevance of nonspecific therapeutic effects in survivorship care settings where safe symptom relief is valued.

Another important consideration when interpreting these findings is the reliance on patient-reported outcome measures, including the CIPNAT, FACT/GOG-Ntx, VAS, and SF-MPQ-2. Although these validated instruments capture clinically meaningful symptom burden, they are inherently susceptible to ascertainment bias when blinding is incomplete. In this review, one study demonstrating significant benefit compared topical henna with standard care and therefore did not blind participants, potentially increasing the risk of biased self-assessment (13). However, another positive study employed a double-blind, placebo-controlled design (15), whereas several blinded studies did not demonstrate statistically significant between-group differences (14, 17). Accordingly, although inadequate blinding may have influenced individual study findings, the available evidence is insufficient to establish a consistent relationship between blinding and treatment efficacy. Future RCTs should continue to incorporate rigorous placebo-controlled designs together with objective assessments of peripheral nerve function to minimize the potential for ascertainment bias.

The observed clinical signals for topical henna and *Costus* species ointment are supported by plausible biological mechanisms that align with current understanding of CIPN pathophysiology (**Figure 3**). Although the initiating neurotoxic mechanisms differ among chemotherapeutic agents, they converge on common downstream pathological processes including mitochondrial dysfunction, oxidative stress, neuroinflammation, ion channel dysregulation, impaired axonal transport and distal axonal degeneration (6). These processes contribute to hyperexcitability within peripheral sensory neurons, leading to spontaneous firing, hyperalgesia, and persistent neuropathic symptoms while also promoting the development of central sensitization (23). Consequently, botanical compounds possessing antioxidant and anti-inflammatory properties may theoretically mitigate these convergent molecular pathways irrespective of the offending chemotherapeutic agent.

**Figure 3:**
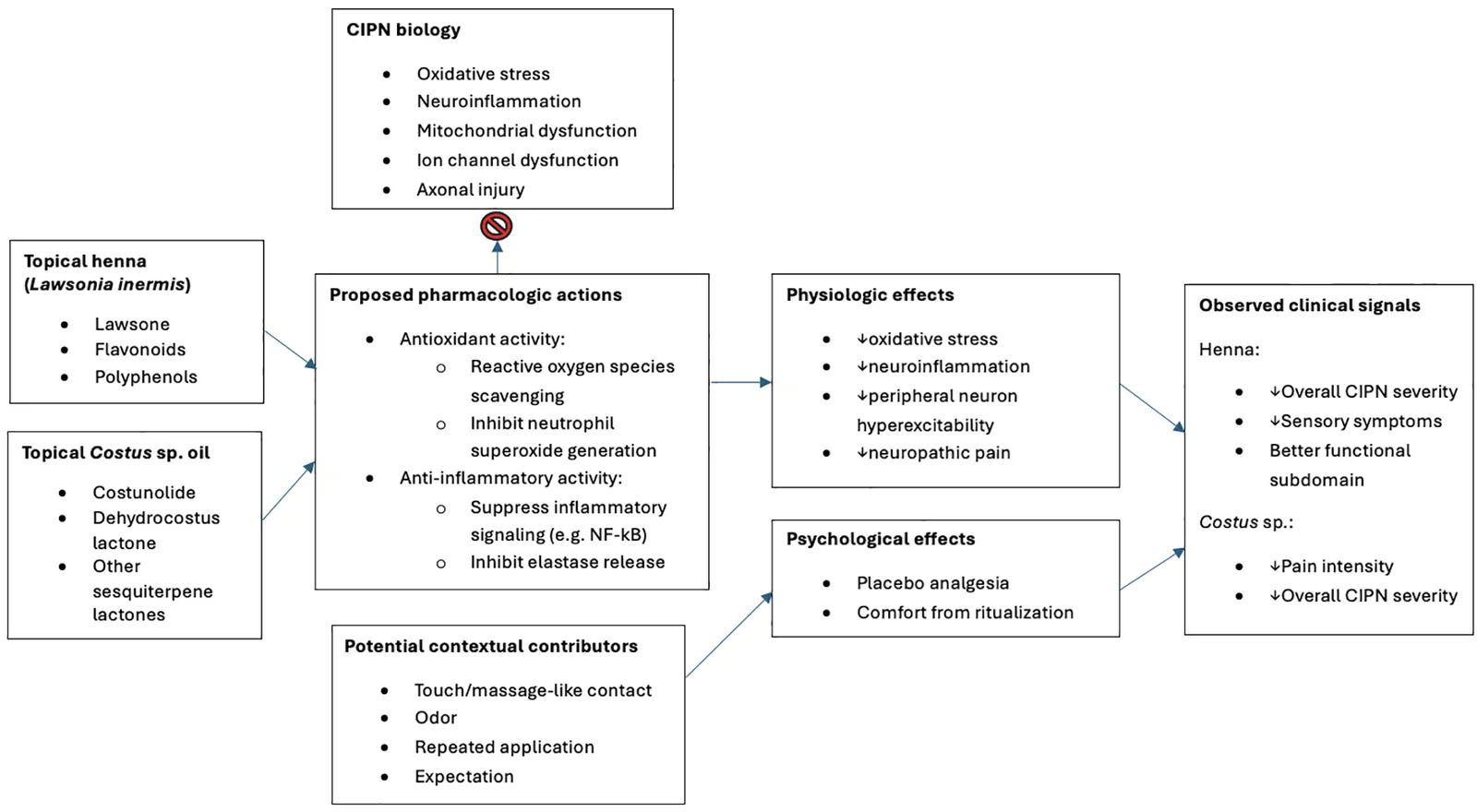
Proposed conceptual framework for the effects of topical henna and *Costus* sp. on CIPN.

The phytochemistry of *Lawsonia inermis* is dominated by lawsone together with flavonoids, tannins, and other polyphenolic compounds that possess antioxidant and anti-inflammatory activity (24). Experimental studies demonstrate that these constituents scavenge reactive oxygen species and inhibit neutrophil superoxide generation and elastase release (25, 26), thereby reducing oxidative stress and inflammatory responses implicated in peripheral nerve injury. Given the central role of oxidative stress and neuroinflammation in the pathogenesis of CIPN, these mechanisms provide a biologically plausible explanation for the selective improvements observed in sensory and functional CIPNAT domains (13). Similarly, *Costus* species contain sesquiterpene lactones, particularly costunolide and dehydrocostus lactone, which exhibit antioxidant and anti-inflammatory properties in experimental models (27, 28). Preclinical studies suggest that these compounds suppress NF-kB-mediated inflammatory signaling, reduce oxidative injury and attenuate neuroinflammation (29–31). These experimental findings offer a mechanistic rationale for the reduction in neuropathic pain observed in the pilot trial (15).

Despite these biologically plausible mechanisms, an important gap remains. CIPN primarily affects dorsal root ganglion neurons and distal peripheral axons (32), but the pharmacokinetics of topical herbal preparations remain poorly understood. No included study evaluated tissue penetration, biomarker changes, pharmacokinetics, or neurophysiologic endpoints capable of demonstrating that topically applied phytochemicals reached dorsal root ganglia or peripheral nerves at therapeutically meaningful concentrations. Consequently, although the observed clinical benefits are consistent with the proposed pharmacologic mechanisms, direct neuroprotective effects remain unconfirmed.

The findings of this review provide several insights that inform future clinical research. First, study populations varied considerably with respect to cancer type and chemotherapy regimen. Because the pathophysiology and clinical course of CIPN differ depending on the causative chemotherapy agent, future trials may benefit from focusing on more homogenous populations. Second, the dosing regimens and herbal formulations differed widely. Standardization of herbal preparations and preliminary dosage studies may be necessary to determine optimal concentrations and application schedules prior to conducting larger efficacy trials. The use of standardized formulations and dosing regimens would also facilitate comparisons across studies and improve the feasibility of future meta-analyses.

Outcome measurement is another crucial area for improvement. All included trials relied primarily on patient-reported outcome measures, including the CIPNAT, FACT/GOG-Ntx, VAS, and SF-MPQ-2. While these instruments capture the subjective burden of neuropathy symptoms, they remain susceptible to ascertainment bias, particularly when participant blinding is incomplete. Future studies should complement patient-reported outcomes with objective laboratory assessments such as quantitative sensory testing or nerve conduction studies to fully reflect changes in peripheral nerve function. These objective outcomes could provide valuable insight into whether topical herbal medicines produce measurable physiologic improvements in nerve function. Integration of biomarkers may further clarify whether observed benefits reflect analgesic effects, modulation of neuroinflammation, or other peripheral processes. Additionally, chemotherapy adherence is a clinically meaningful outcome that has not been assessed in existing trials. Given that CIPN often leads to chemotherapy dose reduction or discontinuation, future studies should consider whether the modification of CIPN symptoms by topical herbal medicine allows patients to maintain planned chemotherapy dose intensity.

### Strengths and limitations

This review focused exclusively on topical herbal medicines delivered as single-modality interventions for CIPN, thereby minimizing confounding factors from heat therapy, aromatherapy, or other multimodal traditional practices, and providing a focal synthesis of an emerging area of integrative oncology. We employed a comprehensive search strategy across multiple databases and conducted dual independent screening and standardized risk-of-bias assessment. Restricting inclusion to RCTs that used validated neuropathy measures strengthens internal validity, despite the limited number of studies.

We also acknowledge several limitations. First, the review protocol was not prospectively registered in a public database such as PROSPERO or INPLASY. Although the review followed PRISMA guidelines and predefined eligibility criteria, the lack of protocol registration may reduce transparency and introduced the potential for reporting bias. Second, only studies published in English were included, which may have resulted in the exclusion of relevant evidence published in other languages and introduced language bias. Third, the number of eligible studies was small, and most trials enrolled relatively few participants, limiting statistical power and generalizability. Fourth, substantial heterogeneity existed across intervention protocols, chemotherapy regimens, and outcome instruments, which precluded quantitative meta-analysis. Fifth, some trials had unclear or high risk of bias related to blinding or outcome assessment, and most relied primarily on subjective self-reported measures of neuropathy symptoms. Finally, the short duration of most studies limits conclusions regarding the long-term sustainability of treatment effects.

## Conclusion

This systematic review identifies limited but suggestive evidence that selected topical herbal medicines, particularly henna and *Costus* species oil ointment, may improve CIPN severity or neuropathic pain in specific clinical settings. Although biologically plausible mechanisms support these findings, the current evidence remains preliminary. Rigorously designed RCTs are needed before topical herbal therapies can be incorporated into evidence-based management for CIPN.

## Data Availability

The original contributions presented in the study are included in the article/**Supplementary Material**. Further inquiries can be directed to the corresponding author.
