# Supplementary material for: Topical herbal medicine as treatment for chemotherapy-induced peripheral neuropathy: a systematic review of randomized controlled trials
Source: Front Oncol. 2026 Sep 28;16:1920042. doi: 10.3389/fonc.2026.1920042 (PMC13654661; doi:10.3389/fonc.2026.1920042)
Supplement: Supplementary file 1 [file Table1.docx]

**Topical Herbal Medicine as Treatment for Chemotherapy-Induced Peripheral Neuropathy: A Systematic Review of Randomized Controlled Trials**

Carrie Shao^1^; Cassie Shao^2^; Andrew Haggarty^3^; Mingxiao Yang^4^; Ting Bao^5^

**Supplemental materials:**

**Search Strategy**

1. Ovid Medline

| 1 | ("5-Hydroxytryptophan" or "2-Amino-3-(5-hydroxy-1H-Indol-3-yl)-Propanoic Acid" or "L-5 Hydroxytryptophan" or "5 Hydroxy-Tryptophan" or "5 Hydroxy-Tryptophane" or "5-Hydroxytryptophane" or "5-Hydroxy L-Tryptophan" or "5-Hydroxy L-Tryptophane" or "5-Hydroxy Tryptophan" or "5-L-Hydroxytryptophan" or "L-5 HTP" or "L-5-Hydroxytryptophan" or "L-5-Hydroxytryptophane" or "Oxitriptan" or "714-X" or "Abscisic Acid" or "Acai" or "Euterpe oleracea" or "E oleracea" or "Euterpe badiocarpa" or "E badiocarpa" or "Assai" or "Baie de Palmier Pinot" or "Cabbage Palm" or "Chou Palmiste" or "Palmier d Acai" or "Agrimony" or "A eupatoria" or "Agrimone" or "Agrimonia" or "Aigremoine" or "A Eupatoire" or "Church Steeples" or "Churchsteeples" or "Cockeburr" or "Cocklebur" or "Da Hua Long Ya Cao" or "Eupatoire-des-Anciens" or "Francormier" or "Herba Agrimoniae" or "Herbe-de-Saint-Guillaume" or "Philanthropos" or "Soubeirette" or "Sticklewort" or "The des Bois" or "The du Nord" or "Toute-Bonne" or "Aikido" or "Gendai Budo" or "Kata" or "Aiyishu" or "Alcoholics Anonymous" or "Alexander Technique" or "Alexander Proprioception" or "Technique Alexander" or "Tecnica Alexander" or "Allicin" or "Aloe" or "A vera" or "A barbadensis" or "A indica" or "A africana" or "A arborescens" or "A natalenis" or "A frutescens" or "A ucriae" or "A ferox" or "A supralaevis" or "A perryi" or "A spicata" or "A Capensis" or "A Perfoliata" or "Aloes" or "Burn Plant" or "Chritkumari" or "Elephant's Gall" or "Gel de la Feuille d'Aloes" or "Ghee-Kunwar" or "Ghi-Kuvar" or "Ghrita-Kumari" or "Gvar Patha" or "Hsiang-Dan" or "Kanya" or "Kumari" or "Latex d'Aloes" or "Lily of the Desert" or "Lu-Hui" or "Miracle Plant" or "Plant of Immortality" or "Plante de l Immortalite" or "Plante de la Peau" or "Plante de Premiers Secours" or "Plante Miracle" or "Plantes des Brulures" or "Sabila" or "Alpha-Linolenic Acid" or "Acide Alpha-Linolenique" or "Acido Alfa Linolenico" or "Acide Gras Essentiel" or "Acide Linolenique" or "Acide Gras N3" or "Acide Gras Polyinsature N3" or "Linolenic Acid" or "Omega 3" or "Omega-3" or "Linum usitatissimum" or "L usitatissimum" or "Linum crepitans" or "L crepitans" or "Linum humile" or "L humile" or "Aceite de Linaza" or "Acide Alpha-Linolenique" or "Acide Gras N-3" or "Aliviraaii" or "Alasi" or "Echter Lein" or "Flachs" or "Flachssamen" or "Graine De Lin" or "Huile de Lin" or "Kattan" or "Keten" or "Lin" or "Linho" or "Lino" or "Linseed Oil" or "Malsag" or "Saatlein" or "Ta Ma" or "Tisii" or "Alpha-Lipoic Acid" or "1,2-dithiolane-3-pentanoic acid" or "1,2-dithiolane-3-valeric acid" or "6,8-thioctic acid" or "5-(1,2-dithiolan-3-yl) valeric acid" or "6,8-dithiooctanoic acid" or "A-Lipoic Acid" or "Acetate Replacing Factor" or "Acide Alpha-Lipoique" or "Acide DL-Alpha- Lipoique" or "Acide Lipoique" or "Acide Thioctique" or "Acide 1,2-dithiolane-3-pentanoique" or "Acide 1,2-dithiolane-3-valerique" or "Acide 5 Valerique (1,2-dithiolan-3-yl)" or "Acide 6,8-dithiooctanoique" or "Acide 6,8-Thioctique" or "Acido Alfa Lipoico" or "Biletan" or "DHLA" or "Dihydrolipoic acid" or "Extrait d'acide Alpha-Lipoique" or "Lipoic Acid" or "Lipoicin" or "R-ALA" or "R-Alpha-Lipoic Acid" or "1,2-Dithiolane-3-Pentanoic Acid" or "R-Dithiolane-3-Pentanoic Acid" or "R-Lipoic Acid" or "RS-Alpha-Lipoic Acid" or "S- Alpha-Lipoic Acid" or "S-Lipoic Acid" or "Sodium-R-Lipoate" or "Thioctacid" or "Thioctan" or "Thioctic Acid" or "Alternative Medicine" or "Alternative Therap*" or "Alternative Approach" or "Alternative Health" or "Amino Acid Therap*" or "Amma Therap*" or "Ammotherap*" or "Ancient Medicine" or "Androstenedione" or "4-androstene-3,17-dione" or "Androst-4-ene-3,17-dione" or "4-Androstene 3, 17-dione" or "Androstene" or "Androstenediona" or "Anma" or "Anthocyanin" or "Anthrophysical Medicine" or "Anthroposophic Medicine" or "Antineoplastons" or "3-Phenylacetylamino-2,6-piperidinedione" or "Phenylacetate" or "Phenylacetylglutamine" or "Phenylacetylisoglutamine" or "Antineoplaston A" or "Antineoplaston A1" or "Antineoplaston A10" or "Antineoplaston A10-1" or "Antineoplaston A2" or "Antineoplaston A3" or "Antineoplaston A4" or "Antineoplaston A5" or "Antineoplaston AS2-1" or "Antineoplaston AS2-5" or "Antineoplaston AS5" or "Antineoplaston Ch" or "Antineoplaston F" or "Antineoplaston H" or "Antineoplaston K" or "Antineoplaston L" or "Antineoplaston O" or "Antioxidant" or "Apitherap*" or "Apipuncture" or "Apis Mellifera Venom" or "Apis Venenum Purum" or "Apiterapia" or "Api-Therap*" or "Api-Treatment" or "Apitherapie" or "Api-Venin-Therapie" or "Bee Sting Therap*" or "Bee Therap*" or "Bee Treatment" or "Bee Venom Therap*" or "Therapie des Abeilles" or "Therapie par Venin d'Abeille" or "Traitement par Piqures d'Abeilles" or "Arachidonic Acid" or "Aristolochia" or "Aristoloche" or "Aristoloquia" or "Birthwort").ti,ab,kf. | 494936 |
| --- | --- | --- |
| 2 | ("Guan Mu Tong" or "Guang Fang Ji" or "Pelican Flower" or "Poison de Terre" or "Pomerasse" or "Ratelaine" or "Sangree Root" or "Sangrel" or "Sarrasine" or "Serpentaire" or "Serpentaria" or "Snakeroot" or "Snakeweed" or "Virginia Serpentary" or "Arnica" or "A montana" or "A fulgens" or "A sororia" or "A latifolia" or "A cordifolia" or "A angustifolia" or "A chamissonis" or "Arnikabluten" or "Bergwohlverleih" or "Doronic d'Allemagne" or "Fleurs d'Arnica" or "Kraftwurz" or "Leopard's Bane" or "Mountain Snuff" or "Mountain Tobacco" or "Plantin des Alpes" or "Quinquina des Pauvres" or "Souci des Alpes" or "Tabac des Savoyards" or "Tabac des Vosges" or "Wolf's Bane" or "Wolfsbane" or "Wundkraut" or "Aromatherap*" or "Aroma" or "Aromaterapia" or "Aromatherapie" or "Aromatic Oils" or "Aromatic Therap*" or "Essential Oils" or "Huiles Aromatiques" or "Huiles Essentielles" or "Scent Therap*" or "Traitement par les Essences de Plantes" or "Absinthe du Desert" or "Common Wormwood" or "Common Worm Wood" or "Desert Wormwood" or "Herba Alba" or "Shih" or "Artichoke" or "Cynara cardunculus" or "Cynara scolymus" or "Alcachofa" or "Alcaucil" or "Artichaut" or "Artischocke" or "Cardo" or "Cardon d'Espagne" or "Cardoon" or "Cynara" or "Extrait d'Artichaut" or "Feuille d'Artichaut" or "Gemuseartischocke" or "Kardone" or "Tyosen-Azami" or "Asian Diet" or "Astragaloside" or "Astragalus" or "A membranaceus" or "Phaca membranacea" or "Astragale" or "Astragali" or "Astragalo" or "Beg Kei" or "Bei Qi" or "Buck Qi" or "Huang Qi" or "Huang Se" or "Huanggi" or "Hwanggi" or "Membranous Milkvetch" or "Milk Vetch" or "Mongolian Milk" or "Mongolian Milkvetch" or "Ogi" or "Reglisse Batarde" or "Reglisse Sauvage" or "Ayurveda" or "Ayurvedic Medicine" or "Medecine Ayurvedique" or "Medecine Traditionnelle Asiatique" or "Medecine Traditionnelle Indienne" or "Traditional Asian Medicine" or "Ba Wei Di Huang Wan" or "Shen Qi Wan" or "Bach" or "Batch Flower Remedies" or "Floratherapie" or "Flower Dilutions" or "Flower Essence" or "Flower Remedies" or "Remedes Floraux" or "Balance Technique" or "Balneotherap*" or "Bain de la Mer Morte" or "Bain Mineral" or "Bain de Soufre" or "Bain Thermal" or "Balneological Treatment" or "Balneoterapia" or "Balneotherapeutics" or "Balneotherapie" or "Balneum" or "Bath Therap*" or "Bath Treatment" or "Crenobalneotherap*" or "Dead Sea Baths" or "Low-Dose Radon Hyperthermia Balneo Treatment" or "Mineral Bath" or "Spa Therap*" or "Sulfur Baths" or "Therapeutic Bathing" or "Therapie Thermale" or "Thermal Baths" or "Thermal Mineral Baths" or "Thermal Therap*" or "Traitement par le Bain" or "Water Therap*" or "Laurus nobilis" or "Bay Leaf" or "Bay Tree" or "Daphne" or "Laurier d'Apollon" or "Laurier Noble" or "Laurier-Sauce" or "Laurier Vrai" or "Bear Bile" or "Behaviour Medicine" or "Berberine" or "Berberina" or "Umbellatine" or "Beta-Carotene" or "A-Beta-Carotene" or "Beta-Caroteno" or "Carotenes" or "Carotenoids" or "Provitamin A" or "Provitamine A" or "Bibliotherap*" or "Bilberry" or "Vaccinium myrtillus" or "Airelle" or "Arandano" or "Black Whortles" or "Bleaberry" or "Brimbelle" or "Burren Myrtle" or "Dyeberry" or "Gueule Noire" or "Huckleberry" or "Hurtleberry" or "Mauret" or "Myrtille" or "Myrtilli Fructus" or "Raisin des Bois" or "Trackleberry" or "Whortleberry" or "Wineberry" or "Bitter Orange" or "Citrus amara" or "Citrus bigarradia" or "Citrus vulgaris" or "Aurantii Fructus" or "Aurantii Pericarpium" or "Aurantium" or "Bigarade" or "Chao Zhi Ke" or "Chisil" or "Extrait de Zeste d'Orange" or "Fleur d'Orange Amere" or "Flos Citri Auranti" or "Fructus Aurantii" or "Green Orange" or "Kijitsu" or "Marmalade Orange" or "Meta-Synephrine" or "N-Methyltyramine" or "Naranja Amarga" or "Neroli Oil" or "Norsynephrine" or "Octopamine" or "Orange Amere" or "Orange de Seville" or "Orange Peel Extract" or "Orange Verte" or "Seville Orange" or "Shangzhou Zhiqiao" or "Sour Orange" or "Synephrine" or "Zeste d'Orange Amere" or "Zhi Ke" or "Zhi Qiao" or "Zhi Shi" or "Black Cohosh" or "Actaea racemose" or "Cimicifuga racemose" or "Actaea macrotys" or "Actee a Grappes" or "Actee Noire" or "Aristolochiaceae Noire" or "Baie d'actee" or "Baneberry" or "Black Aristolochiaceae" or "Bugbane" or "Bugwort" or "Cimicaire a grappes" or "Cimicifuga" or "Cimicifuge" or "Cohosh Negro" or "Cohosh Noir" or "Cytise" or "Macrotys" or "Phytoestrogen" or "Phytoestrogene" or "Racine de Serpent" or "Racine de Squaw" or "Racine Noire de Serpents" or "Rattle Root" or "Rattle Top" or "Rattlesnake Root" or "Rattleweed" or "Rhizoma Cimicifugae" or "Sheng Ma" or "Squaw Root" or "Piper nigrum" or "Hu Jiao" or "Kali Mirchi" or "Kosho" or "Marich" or "Maricha" or "Miris" or "Peber" or "Peper" or "Peppar" or "Pepper" or "Peppercorn" or "Pfeffer" or "Pimenta" or "Pimienta" or "Pipar" or "Piper" or "Piperine" or "Pippuri" or "Poivre" or "Poivrier" or "Vellaja" or "Black Seed" or "Nigella sative" or "Ajenuz" or "Aranuel" or "Baraka" or "Charnuska" or "Cheveux de Venus" or "Cominho Negro" or "Comino Negro" or "Fennel Flower" or "Fitch" or "Graine de Nigelle" or "Graine Noire" or "Habatul Sauda" or "Habbatul Baraka" or "Kalajaji" or "Kalajira" or "Kalonji" or "Ketsah" or "La Grainer Noire" or "Love in a Mist" or "Mugrela" or "Nielle" or "Nigelle de Crete" or "Nigelle Cultivee" or "Nutmeg Flower" or "Poivrette" or "Roman-Coriander" or "Schwarzkummel" or "Seed of Blessing" or "Siyah Dane" or "Shoniz" or "Small Fennel" or "Toute Epice" or "Upakuncika" or "Blueberry" or "Blueberries" or "Vaccinium angustifolium" or "V angustifolium" or "Vaccinium brittonii" or "Vaccinium lamarckii" or "Vaccinium pensylvanicum" or "Vaccinium virgatum" or "Vaccinium ashei" or "Vaccinium amoenum" or "Vaccinium corymbosum" or "Vaccinium constablaei" or "Vaccinium pallidum" or "Vaccinium altomontanum" or "Vaccinium vacillans" or "V brittonii" or "V lamarckii" or "V pensylvanicum" or "V virgatum" or "V ashei" or "Vamoenum" or "V corymbosum" or "V constablaei" or "V pallidum" or "V altomontanum" or "V vacillans" or "Arandano" or "Bleuet" or "Bleuets" or "Myrtille" or "Rubel" or "Tifblue" or "Bobath" or "Borage" or "B officinalis" or "Bee Plant" or "Beebread" or "Borago" or "Borraja" or "Bourrache" or "Burage" or "Burrage" or "Common Bugloss" or "Cool Tankard" or "Langue de Boeuf" or "Ox's Tongue" or "Pain-des-Abeilles" or "Starflower" or "Talewort" or "Boron" or "Atomic number 5" or "Acide Borique" or "Anhydride Borique" or "B symbole chimique" or "Borate" or "Borates" or "Boric Acid" or "Boric Anhydride" or "Boric Tartrate" or "Numero Atomique 5" or "Botanicals" or "Bowen Technique" or "Bowen Manipulative Therap*" or "Bowen Therap*" or "Methode Bowen" or "Tecnica Bowen" or "Technique Bowen" or "Technique de Bowen" or "Therapeute en Bowen" or "Therapie Manuelle" or "Bromelain" or "A comosus" or "Pineapple" or "A duckei" or "A sativus" or "Bromelia ananas" or "Bromelia comosa" or "B comosa" or "Ananas" or "Bromelaine" or "Bromelains" or "Bromelainum" or "Bromelin" or "Bromelina" or "Bromeline" or "Enzyme d'Ananas" or "Extrait d'Ananas" or "Fruit Bromelain" or "Protease" or "Brown Peterson Technique" or "Buddhist Tantric Practice" or "Burdock" or "A minus" or "A tomentosum" or "Arctium" or "Bardana" or "Bardana-Minor" or "Bardanae Radix" or "Bardane" or "Beggar's Buttons" or "Burr Seed" or "Clotbur" or "Cocklebur" or "Cockle Buttons" or "Fox's Clote" or "Glouteron" or "Grande Bardane" or "Great Bur" or "Great Burdocks" or "Happy Major" or "Hardock" or "Harebur" or "Lappa" or "Love Leaves" or "Niubang" or "Niu Bang Zi" or "Orelha-de-Gigante" or "Personata" or "Philanthropium" or "Rhubarbe du Diable" or "Thorny Burr" or "Butterbur" or "Petasites" or "P hybridus" or "P officinalis" or "Tussilago hybrida" or "Blatterdock" or "Bog Rhubarb" or "Bogshorns" or "Butter Bur" or "Butter-Dock" or "Butterfly Dock" or "Capdockin" or "Chapeliere" or "Contre-Peste" or "Exwort" or "Flapperdock" or "Grand Bonnet" or "Langwort" or "Pestwurz" or "Petasite" or "P Hybride" or "P Officinal" or "P Vulgaire" or "P Vulgaris" or "Petasitidis Folium" or "Petasitidis Hybridus" or "Petasitidis Rhizoma" or "Plague Root" or "Umbrella Leaves" or "P Folium" or "P Hybridus" or "P Rhizoma" or "Bwiti" or "Calcium" or "Bone Meal" or "Calcarea Carbonica" or "Calcarea Phosphorica" or "Calcio" or "Coquilles d'Huitres Moulues" or "Coquilles d'oeuf" or "Di-Calcium Phosphate" or "Dolomite" or "Hydroxyapatite" or "Microcrystalline Hydroxyapatite" or "OsseinHydroxyapatite" or "Oyster Shell" or "Phosphate Tricalcium" or "Poudre d'os" or "Tricalcium Phosphate" or "Calendula" or "C officinalis" or "Calendule" or "Fleur de Tous les Mois" or "Gold-Bloom" or "Holligold" or "Marigold" or "Marybud" or "Souci des Champs" or "Souci des Jardins" or "Souci des Vignes" or "Souci Officinal" or "Zergul" or "Calorie Control" or "Cannabis" or "Cannabinoids" or "C sativa" or "Anashca" or "Banji" or "Bhang" or "Blunt" or "Charas" or "Esrar" or "Gaga" or "Ganga" or "Grass" or "Haschisch" or "Hashish" or "Herbe" or "Huo Ma Ren" or "Marie-Jeanne" or "Mariguana" or "Marihuana" or "Marijuana" or "Mary Jane" or "Sawi" or "Sinsemilla" or "Weed" or "Cannabidiol" or "Tetrahydrocannabinol" or "Capsaicin" or "Capsicum" or "C frutescens" or "C annuum" or "C chinense" or "C baccatum" or "C pubescens" or "C minimum" or "African Chillies" or "Aji" or "Capsaicine" or "C Oleoresin" or "Cayenne" or "Chili" or "Chilli" or "Chillies" or "Cis-capsaicin" or "Civamide" or "Goat's Pod" or "Grains of Paradise").ti,ab,kf. | 952981 |
| 3 | ("Ici Fructus" or "Katuvira" or "Mexican Chilies" or "Mirchi" or "Paprika" or "Pili-pili" or "Piment Enrage" or "Piment Fort" or "Piment-oiseau" or "Pimento" or "Trans-Capsaicin" or "Zucapsaicin" or "Zucapsaicine" or "Caraway" or "Carum carvi" or "Carum velenovskyi" or "C carvi" or "C velenovskyi" or "Alcaravea" or "Anis Canadien" or "Anis des Pres" or "Anis des Vosges" or "Carraway" or "Carvi" or "C Commun" or "C Fructus" or "Faux Anis" or "Haravi" or "Jeera" or "Jira" or "Karwiya" or "Krishan Jeeraka" or "Krishnajiraka" or "Kummel" or "Kummich" or "Semen Cumini Pratensis" or "Shahijra" or "Shiajira" or "Wiesen-Feldkummel" or "L-Carnitine" or "3-carboxy-2-hydroxy-N,N,N-trimethyl-1-propanaminium inner salt" or "(3-carboxy2-hydroxypropyl) trimethylammonium hydroxide inner salt" or "B-hydroxy-Ntrimethyl aminobutyric acid" or "Beta-hydroxy-gammatrimethylammonium butyrate" or "L-3-hydroxy-4-(trimethylammonium)-butyrate" or "(R)-(3-carboxy-2 hydroxypropyl)trimethylammonium hydroxide" or "(R)-3-hydroxy-4-trimethylammonio-butyrate" or "3-hydroxy-4-Ntrimethylaminobutyrate" or "Aminocarnitine" or "Beta-hydroxyl-gamma-tributyl aminobutyrate" or "B(t) Factor" or "Carnitine" or "Carnitor" or "D-Carnitine" or "DLCarnitine" or "Facteur B(t)" or "L-b-hydroxy-c-N-trimethylaminobutyric acid" or "L-Carnitina" or "Levocarnitine" or "Vitacarn" or "Vitamin B(t)" or "Vitamine B(t)" or "Acetyl-L-Carnitine" or "2-(acetyloxy)-3-carboxy-N,N,N-trimethyl-1-propanaminium inner salt" or "(3-carboxy-2-hydroxypropyl)trimethylammonium hydroxide inner salt acetate" or "Acetil-L-Carnitina" or "Acetyl-LCarnitine HCl" or "Acetyl L-Carnitine Hydrochloride" or "Acetyl Carnitine" or "Acetyl-Carnitine" or "Acetyl-Levocarnitine" or "Aminocarnitine" or "Carnitine Acetyl Ester" or "Dihydrochlorure d'Acetyl-L-Carnitine Arginate" or "Gamma-Trimethyl-BetaAcetylbutyrobetaine" or "L-Acetylcarnitine" or "Levacecarnine" or "N-Acetyl-Carnitine" or "N-AcetylCarnitine Hydrochloride" or "N-Acetyl-L-Carnitine" or "Vitamin B(t) Acetate" or "Cartilage Supplement" or "Shark Cartilage" or "Squalus acanthias" or "Spiny Dogsh Shark" or "Sphyrna lewini" or "Scalloped Hammerhead Shark" or "AE-941" or "Cartilage de Requin" or "Cartilago de Tiburon" or "Collagene Marin" or "Extrait de Cartilage de Requin" or "Liquide de Cartilage Marin" or "Marine Collagen" or "Marine Liquid Cartilage" or "Neovastat" or "Poudre de Cartilage de Requin" or "Bovine Cartilage" or "Antitumor Angiogenesis Factor" or "Bovine Tracheal Cartilage" or "Cartilage Tracheal de Bovins" or "Cartilago Bovino" or "Catrix" or "Catrix-S" or "Collagen Bovine" or "Collagene Bovin" or "Glycosaminoglycan Polysulphuric Acid Complex" or "Psoriacin" or "Psoriacin-T" or "Rumalon" or "Casein Free Diet" or "Catgut Implantation" or "Cat's Claw" or "Uncaria guianensis" or "Uncaria tomentosa" or "U guianensis" or "U tomentosa" or "Griffe Du Chat" or "Liane du Perou" or "Life-Giving Vine of Peru" or "Peruvian Liana" or "Samento" or "Una De Gato" or "Cellasene" or "Cetyl Myristoleate" or "Chamomile" or "Chamomilla recutita" or "Camomele" or "Camomilla" or "Camomille" or "Chamomile" or "Chamomilla" or "Echte Kamille" or "Feldkamille" or "Fleur de Camomile" or "Kamillen" or "Kleine Kamille" or "Manzanilla" or "M Alemana" or "Matricaire" or "OEil du Soleil" or "Pin Heads" or "Chamaemelum nobile" or "Ormenis nobilis" or "C nobile" or "A nobilis" or "O nobilis" or "Anthemis" or "A Odorante" or "A nobilis" or "Babounge" or "Babuna Ke Phool" or "Chamomillae Ramane Flos" or "Flores Anthemidis" or "F Anthemidis" or "Grosse Kamille" or "Ground Apple" or "Manzanilla" or "M Romana" or "Romische Kamille" or "Whig Plant" or "Chanting" or "Cheirology" or "Chelated Minerals" or "Mineral-Amino Acid Complex" or "Bore Chelate" or "Chelated Boron" or "Chelated Cobalt" or "Chelated Iron" or "Chelated Molybdenum" or "Chelated Potassium" or "Chelated Selenium" or "Chelated Trace Minerals" or "Chelated Vanadium" or "Chelated Zinc" or "Cobalt Chelate" or "Cuivre Chelate" or "Fer Chelate" or "Minerales Quelados" or "Mineraux Chelates" or "Molybdene Chelate" or "Potassium Chelate" or "Selenium Chelate" or "Vanadium Chelate" or "Zinc Chelate" or "Chitosan" or "Ascorbate de Chitosane" or "Chitosan-N-Acetylcysteine" or "Deacetylated Chitin" or "Enzymatic Polychitosamine Hydrolisat" or "Poly-D-Glucosamine" or "Poly-N-Acetyl-Glucosamine" or "Quitosano" or "Sulfated N-Carboxymethylchitosan" or "Sulfated O-Carboxymethylchitosan" or "Choline" or "Trimethylethanolamine" or "beta-hydroxyethyl" or "trimethylammonium hydroxide" or "Colina" or "Facteur Lipotropique" or "Hydroxyde de Trimethylammonium" or "beta-hydroxyethyl" or "Intrachol" or "L-Choline" or "Lipotropic Factor" or "Methylated Phosphatidylethanolamine" or "Trimethylethanolamine" or "Chondroitin Sulfate" or "Chondroitin 4-sulfate" or "Chondroitin 4- and 6-sulfate" or "Chondroitin Polysulfate" or "Chondroitin Sodium Sulfate" or "Chondroitin Sulphate" or "Chondroitine" or "Chondroitine Sulfate A" or "Chondroitine Sulfate B" or "Chondroitine Sulfate C" or "Chondroitine 4-Sulfate" or "Chondroitine 4- et 6-Sulfate" or "Condroitin" or "Galactosaminoglucuronoglycan Sulfate" or "Poly-(1->3)-N-Acetyl-2-Amino-2- Deoxy-3-O-Beta-D-Glucopyranurosyl-4-(or 6-)" or "Polysulfate de Chondroitine").ti,ab,kf. | 139529 |
| 4 | ("Radix Angelicae Sinensis" or "Tan Kue Bai Zhi" or "Tang Kuei" or "Tanggwi" or "Drama Therapy" or "Dream Therapy" or "Dukun" or "Ear Candl*" or "Auricular Candles" or "Auriculotherapie" or "Bougie Creuse" or "Bougie Hopi" or "Bougie d Oreille" or "Chandelles Auriculaires" or "Conage d Oreille" or "Ear Candle Therapy" or "Ear Candle Treatment" or "Ear Coning" or "Terapia de Vela en el Oido" or "Therapie Auriculaire Thermale" or "Thermal-Auricular Therapy" or "Echinacea" or "Brauneria angustifolia" or "Brauneria pallida" or "Rudbeckia pallida" or "Brauneria purpurea" or "Helichroa purpurea" or "Rudbeckia purpurea" or "American Cone Flower" or "Black Sampson" or "Black Susans" or "Comb Flower" or "Coneflower" or "Echinaceawurzel" or "Echinacee" or "Equinacea" or "Fleur A Herisson" or "Hedgehog" or "Igelkopfwurzel" or "Indian Head" or "Kansas Snakeroot" or "Narrow-Leaved Echinacea" or "Narrow-Leaved Purple Coneflower" or "Narrow-leaved Purple Cone Flower" or "Pale Coneflower" or "Pale Flower Echinacea" or "Pale Purple Coneflower" or "Purple Cone flower" or "Purpursonnenhutkraut" or "Purpursonnenhutwurzel" or "Racine D echininacea" or "Red Sunflower" or "Rock-Up-Hat" or "Roter Sonnenhut" or "Rudbeckie Pourpre" or "Schmallblaettrige Kegelblumenwurzel" or "Schmallblaettriger Sonnenhut" or "Scurvy Root" or "Snakeroot" or "Sonnenhutwurzel" or "Eclectic Medicine" or "Edetic Acid" or "Edetate" or "Effleurage" or "Eicosapentaenoic Acid" or "20:5n-3" or "Acide Eicosapentaenoique" or "Acide Ethyle-Eicosapentaenoique" or "Acide Gras Essentiel" or "Acide Gras d Huile de Poisson" or "Acide Gras N-3" or "Acide Gras Omega 3" or "Acide Gras Polyinsature" or "Acide Gras W3" or "Acido Eicosapentaenoico" or "Eicosapentanoic Acid" or "Ethyl Eicosapentaenoic Acid" or "Ethyl-EPA" or "Icosapent Ethyl" or "PUFA" or "Elemental Diet" or "Ellagic Acid" or "Acide Ellagique" or "Acido Elagico" or "Gallogen" or "Emodin" or "Emotional Freedom Technique" or "Tapping" or "Bioenergie" or "Biofield Therapies" or "Environmental Medicine" or "Enzyme Therapy" or "Ephedra" or "Alcaloide d Ephedrine" or "Belcho" or "Cao Mahuang" or "Chinese Joint-Fir" or "Cao Ma-Huang" or "Desert Herb" or "Efedra" or "Ephedre" or "Ephedrine" or "Epitonin" or "Herbal Ecstasy" or "Indian Jointfir" or "Joint Fir" or "Ma Huang" or "Mahuanggen" or "Popotillo" or "Raisin de Mer" or "Sea Grape" or "Teamsters Tea" or "The de Desert" or "Yellow Astringent" or "Yellow Horse" or "Zhong Mahuang" or "Esoteric Therapy" or "Essaic" or "Essence Therapy" or "Estrogen Supplement" or "Ethnomedicine" or "Ethylenediaminetetraacetic Acid" or "Evening Primrose" or "Oenothera biennis" or "Oenothera muricata" or "Oenothera rubricaulis" or "Oenothera suaveolens" or "Onagra biennis" or "Aceite de Onagra" or "Acide Cis-linoleique" or "Cis-Linoleic Acid" or "Evening Star" or "Fever Plant" or "Herbe-aux-anes" or "Huile de Graines d Onagre" or "Huile D Onagre" or "Huile de Primerose" or "Huile de Primevere Vesperale" or "Jambon de Jardinier" or "Jambon du Paysan" or "Kings Cureall" or "Mache Rouge" or "Night Willow-Herb" or "OEnothere" or "Onagraire" or "Onagre Bisannuelle" or "Onagre Commune" or "Primevere du Soir" or "Primrose" or "Scabish" or "Scurvish" or "Sun Drop" or "Exercise" or "Physical Therapy" or "Experience Based Medicine" or "Eye Movement Desensitization and Reprocessing" or "Fasting" or "Caloric Restriction" or "Energy Restriction" or "Intermittent Energy Restriction" or "Intermittent Severe Energy Restriction" or "Time-Restricted Feeding" or "Total Caloric Desistance" or "Zero Calorie Diet" or "Fat-Restricted Diet" or Fatty acid* or "Acides Gras Cetylated" or "Acides Gras Cetyles" or "Acides Gras Esterises").ti,ab,kf. | 881097 |
| 5 | ("Acides Gras Mono-Insatures Cetyles" or "Acidos Grasos Cetilados" or "Cerasomal-cis-9-cetylmyristoleate" or "Cetyl Decanoate" or "Cetyl Laurate" or "Cetyl Laureate" or "Cetyl Myristate" or "Cetyl Myristoleate" or "Cetyl Oleate" or "Cetyl Palmitate" or "Cetyl Palmitoleate" or "Cetyl Stearate" or "Cetylmyristoleate" or "Cis-9-cetylmyristoleate" or "Laureate Cetyl" or "Myristate Cetyl" or "Myristoleate Cetyl" or "Oleate Cetyl" or "Palmitate Cetyl" or "Palmitoleate Cetyl" or "Acides Gras Essentiels N-6" or "Acides Gras Omega-6" or "Acides Gras Polyinsatures" or "Acidos Grasos Omega 6" or "Huiles d Omega 6" or "Omega 6 Oils" or "Fusui" or "Phong-Thuy" or "Pung-Su" or "Fenugreek" or "Trigonella foenum-graecum" or "Alholva" or "Bird's Foot" or "Bockshornklee" or "Bockshornsame" or "Chandrika" or "Fenogreco" or "Fenugrec" or "Foenugraeci Semen" or "Foenugreek" or "Greek Clover" or "Greek Hay" or "Greek Hay Seed" or "Hu Lu Ba" or "Methi" or "Methika" or "Medhika" or "Senegrain" or "Senegre" or "Trigonella" or "Trigonelle" or "Woo Lu Bar" or "Feverfew" or "Tanacetum parthenium" or "Chrysanthemum parthenium" or "Chrysanthemum praealtum" or "Leucanthemum parthenium" or "Matricaria eximia" or "Matricaria parthenium" or "Pyrethrum parthenium" or "Altamisa" or "Bachelor's Buttons" or "Chrysantheme Matricaire" or "Featerfoiul" or "Featherfew" or "Featherfoil" or "Flirtwort Midsummer Daisy" or "Grande Camomille" or "Matricaria" or "Partenelle" or "Pyrethre Dore" or "Pyrethre Mousse" or "Santa Maria" or "Tanaceti Parthenii" or "Tanaisie Commune" or "Fish Oil" or "Aceite de Pescado" or "Acides Gras Omega 3" or "Acides Gras N-3" or "Acides Gras Polyinsatures N-3" or "Acides Gras W-3" or "Ethyl Ester" or "Ester Ethylique de l'AEP" or "Ester Ethylique de l'ADH" or "Herring Oil" or "Huile de Foie de Morue" or "Huile de Hareng" or "Huile de Menhaden" or "Huile de Poisson" or "Huile de Saumon" or "Huile de Thon" or "Huile Lipidique Marine" or "Huile Marine*" or "Lipides Marins" or "Marine Lipid Concentrate" or "Marine Lipids" or "Marine Oil" or "Marine Fish Oil" or "Marine Lipid Oil" or "Marine Triglyceride" or "Menhaden Oil" or "Salmon Oil" or "Triglycerides Marins" or "Tuna Oil" or "Tuna Fish Oil" or "Chrysin*" or "5,7-dihydroxy-2-phenyl-4H-chromen-4-one" or "5,7-Chrysin, 5,7-Dihydroxyavone" or "Flavone X" or "Flavonoid*" or "Galangin*" or "Flavanone" or "Diosmin*" or "3', 5, 7-trihydroxy-4'-methoxyavone-7-rhamnoglucoside" or "Bioavonoid*" or "Bioavonoid Complex" or "Bioavonoid Concentrate" or "Bioavonoid Extract" or "Bioavonode* d Agrume*" or "Citrus Bioavones" or "Citrus Bioavonoid*" or "Citrus Bioavonoid Extract" or "Citrus Flavones" or "Citrus Flavonoids" or "Complexe de Bioavonoide*" or "Concentre de Bioavonoide*" or "Diosmetin 7-O-rutinoside" or "Extrait de Bioavonoide*" or "Micronised Puried Flavonoid Fraction" or "Quercetin*" or "3,3',4'5,7-Pentahydroxyavone" or "Bioavonoide* de Citron" or "Flavones de Citron" or "Meletin*" or "Quercetin Dihydrate" or "Sophretin*" or "Hesperidin*" or "Hesperidin Methyl Chalcone" or "Trimethylhesperidin-Chalcon" or "Rutin*" or "3, 3', 4', 5, 7-pentahydroxyavone-3-rhamnoglucoside" or "Eldrin" or "Flavonoides d Agrumes" or "Quercetin-3-Rhamnoglucoside" or "Quercetin*-3-Rutinoside" or "Rutosid*" or "Sclerutin" or "Sophorin" or "Flaxseed" or "Linum usitatissimum" or "Linum crepitans" or "Linum humile" or "Alasi" or "Aliviraaii" or "Echter Lein" or "Flachs" or "Flax" or "Flachssamen" or "Graine de Lin" or "Kattan" or "Keten" or "Leinsamen" or "Lignanes de Lin" or "Lignans" or "Linaza" or "Lini Semen" or "Linho" or "Lino" or "Linseed" or "Lint Bells" or "Linum" or "Malsag" or "Phyto-Oestrogene" or "Saatlein" or "Tisii" or "Winterlien" or "Flor Essence Formula" or "Flower Essence" or "Flower Remedies" or "Folk Medicine" or "Frangula purshiana" or "Free and Easy Wanderer" or "Fringe Medicine" or "Fructans" or "Fructooligosaccharides" or "Chicory Inulin Hydrolysate" or "Complexe d'Oligosaccharide" or "Fructo Oligo Saccharides" or "Fructo-Oligosacaridos" or "Inulin Hydrolysate" or "Oligofructan" or "Oligofructose" or "Oligosaccharides" or "Prebiotic" or "Prebiotique" or "Short Chain Fructo-Oligosaccharides" or "Fu Zheng" or "Fuzheng" or "Galactans" or "Galacto-Oligosaccharides" or "Galactooligosaccharides" or "Gamma Oryzanol" or "Gama Orizanol" or "Gamma-OZ" or "Oryzanol" or "Gamma-Linolenic Acid" or "Acide Gammalinolenique" or "Acide Gamma-Linolenique" or "Acido Gama Linolenico" or "Gammalinolenic Acid" or "Gamolenic Acid" or "(Z,Z,Z)-Octadeca-6,9,12-Trienoic Acid" or "Garcinia" or "Cambogia binucao" or "Cambogia gemmi-guta" or "Cambogia solitaria" or "Garcinia affinis" or "Garcinia cambogia" or "Garcinia sulcata" or "Mangostana cambogia" or "Brindal Berry" or "Brindle Berry" or "Gorikapuli" or "Kankusta" or "Kudam Puli" or "Malabar Tamarind" or "Tamarinier de Malabar" or "Vrikshamla" or "Garlic" or "Allium sativum" or "Ajo" or "Alho" or "Allii Sativi Bulbus" or "Allium" or "Angio D India" or "Camphor Of The Poor" or "Echte Rokkenbolle" or "Knoblauch" or "Lahsun" or "Lasun*" or "Maneul" or "Nectar Of The Gods" or "Ninniku" or "Poor Man's Treacle" or "Rason" or "Rocambole" or "Rockenbolle" or "Rust Treacle" or "Schlangenknoblauch" or "Stinking Rose" or "Suan" or "Thoum" or "Vitlok" or "Genistein Combined Polysaccharide" or "Basidiomycetes Polysaccharide" or "Fermented Genistein" or "Fermented Isoflavone" or "Genistein Polysaccharide" or "Genisteine du Polysaccharide Combine" or "Genisteine Fermentee" or "Isoflavone Combined Polysaccharide" or "Isoflavone Fermentee" or "Polisacaridos Combinados de Genisteina" or "Polysaccharide de Genisteine" or "Polysaccharide des Basidiomycetes" or "Polysaccharide d Isoflavone de Soja" or "Soy Isoflavone Polysaccharide" or "Germander" or "Teucrium chamaedrys" or "Camedrio" or "Chasse-Fievre" or "Cheneau" or "Chenette" or "Germandree" or "Petit Chene" or "Teucrium scordium" or "Chamarras" or "Escordio" or "Germandrina de Agua" or "Gerson Therapy" or "Gerson Diet" or "Gestalt Therapy" or "Ginger" or "Zingiber officinale" or "Amomum zingiber" or "Ardraka" or "Gan Jiang" or "Gingembre" or "Huile Essentielle de Gingembre" or "Imber" or "Jengibre" or "Jiang" or "Kankyo" or "Kanshokyo" or "Nagara" or "Rhizoma Zingiberi*" or "Rhizoma Zingiberis Recens" or "Shen Jiang" or "Sheng Jiang" or "Shoga" or "Shokyo" or "Shunthi" or "Srungavera" or "Sunth" or "Sunthi" or "Vishvabheshaja" or "Zinzeberis" or "Zinziber Officinale" or "Zinziber Officinalis" or "Ginkgo" or "Abricot Argente Japonais" or "Adiantifolia" or "Arbe aux Ecus" or "Arbe aux Quarante Ecus" or "Arbe du Ciel" or "Arbre Fossile" or "Bai Guo Ye" or "Baiguo" or "Fossil Tree" or "Japanese Silver Apricot" or "Kew Tree" or "Maidenhair Tree" or "Noyer du Japon" or "Pei Go Su Ye" or "Salisburia Adiantifolia" or "Yen Xing" or "Yinhsing" or "Ginseng" or "Panax schinseng" or "Guigai" or "Hong Shen" or "Insam" or "Jen-Shen" or "Jinsao" or "Jintsam" or "Korean Panax" or "Mandragore de Chine" or "Ninjin" or "Ninzin" or "Panax Coreen" or "Racine de Vie" or "Renshen" or "Renxian" or "Sheng Shai Shen" or "Panax quinquefolius" or "Baie Rouge" or "Panax Quinquefoli*" or "Red Berry" or "Ren Shen" or "Shang" or "Shi Yang Seng" or "Xi Yang Shen" or "Glucosamine" or "3-Amino-6-(Hydroxymethyl)Oxane-2,4,5-Triol Sulfate" or "(3R,4R,5S,6R)-3-Amino-6-(Hydroxymethyl)Oxane-2,4,5-Triol Hydrochloride" or "2-Acetamido-2-deoxyglucose" or "2-Amino-2-Deoxy-Beta-D-Glucopyranose Hydrochloride" or "2-Amino-2-Deoxy-D-Glucosehydrochloride" or "2-Amino-2-Deoxy-Beta-D-Glucopyranose" or "2-Amino-2-Deoxy-D-Glucose Sulfate" or "Acetylglucosamine" or "GlcNAc" or "Chitosamine" or "Chlorhidrato de Glucosamina" or "Chlorhydrate de Glucosamine" or "D-Glucosamine HCl" or "D-Glucosamine Hydrochloride" or "D-Glucosamine Sulfate" or "D-Glucosamine Sulphate" or "Mono-Sulfated Saccharide" or "NAcetil Glucosamina" or "N-Acetylglucosamine" or "N-Acetyl D-Glucosamine" or "Poly-N-Acetyl Glucosamine" or "Poly-NAG" or "Poly-(1-3)-N-Acetyl-2-Amino-2-Deoxy-3-OBeta-D-Glucopyranurosyl-4-(or 6-) Sul" or "Saccharide Mono-Sulfate" or "Saccharide Sulfate" or "Sulfate de Glucosamine" or "Sulfated Monosaccharide" or "Sulfated Saccharide" or "Sulfato de Glucosamina" or "Glutamine").ti,ab,kf. | 301605 |
| 6 | ("L-(+)-2-Aminoglutaramic acid" or "Acide Glutamique" or "Acide L-(+)-2-Aminoglutaramique" or "Acide L-Glutamique" or "Alanyl-L-Glutamine Dipeptide" or "Glutamate" or "Glutamic Acid" or "Glutamina" or "Glutaminate" or "Levoglutamide" or "Levoglutamine" or "L-Alanyl-L-Glutamine" or "L-Glutamic Acid" or "L-Glutamine" or "N-Acetyl-L-Glutamine" or "(S)-2,5-Diamino-5-oxopentanoic Acid" or "Gluten-Free Diet" or "Wheat-Free Diet" or "Glyconutrients" or "Ambrotose" or "Gluconutrientes" or "Glyconutriments" or "Manapol" or "Grahamism" or "Vitis vinifera" or "Vitis labrusca" or "Activin" or "Calzin" or "Draksha" or "Enocianina" or "Raisins" or "Feuille de Vigne Rouge" or "Flame Seedless" or "Folia Vitis Viniferae" or "Grapes" or "Grapeseed" or "Kali Draksha" or "Leucoanthocyanin" or "Muscat" or "Muskat" or "Oligomeres Procyanidoliques" or "Oligomeric Proanthocyanidins" or "Oligomeric Procyanidins" or "Petite Sirah" or "Proanthocyanidines Oligomeriques" or "Proanthodyn*" or "Procyanidines Oligomeriques" or "Procyanidolic Oligomers" or "Raisin" or "Red Globe" or "Red Malaga" or "Red Vine" or "Sultanas" or "Thompson Seedless" or "Vin Rouge" or "Greater Celandine" or "Chelidonium majus" or "Bai Qu Cai" or "Celandine" or "Celidonia Mayor" or "Chelidonii Herba" or "Grande Chelidoine" or "Grande Eclaire" or "Herbe a Verrues" or "Herbe aux Verrues" or "Parties Aeriennes de la Grande Chelidoine" or "Racine de Chelidoine" or "Rhizome de Chelidoine" or "Schollkraut" or "Swallow Wort" or "Tetterwort" or "Verruguera" or "Green Tea" or "Camellia sinensis" or "Camellia thea" or "Camellia theifera" or "Thea bohea" or "Thea sinensis" or "Thea viridis" or "Benifuuki" or "Constituant Polyphenolique de The Vert" or "Epigallo Catechin Gallate" or "Epigallo-Catechine Gallate" or "Epigallocatechin Gallate" or "Extrait de The Vert" or "Kunecatechins" or "Poly E" or "Polyphenon E" or "Te Verde" or "Tea Extract" or "Tea" or "Yabukita" or "Gso-Ba Rig-Pa" or "Guarana" or "Paullinia cupana" or "Paullinia sorbilis" or "Brazilian Cocoa" or "Cacao Bresilien" or "Guarana Seed Extract" or "Guaranine" or "Guggul" or "Commiphora wightii" or "Commiphora mukul" or "Balsamodendrum wightii" or "Balsamodendrum mukul" or "Devadhupa" or "Gomme Guggul" or "Gomme-Resine de Guggul" or "Guggal" or "Guggulipid*" or "Guggulsterone*" or "Guggulu" or "Guglipid" or "Gugulipid" or "Gum Guggal" or "Indian Bdellium" or "Indian Guggulipids" or "Koushika" or "Mukul Myrrh Tree" or "Palankasha" or "Imagerie Guidee" or "Imagery" or "Imaginacion Guiada" or "Visualisation" or "Visualization" or "Guiling Pa'an Wan" or "Gurah" or "Hair Analysis" or "Hands-On Healing" or "Hawthorn" or "Mespilus laevigata" or "Aubepine" or "Bianco Spino" or "Bois de Mai" or "Cenellier" or "Crataegi Flos" or "Crataegi Folium" or "Crataegi Fructus" or "Crataegus" or "Epine Blanche" or "Epine de Mai" or "Espino Blanco" or "Fructus Crataegi" or "Haagdorn" or "Hagedorn" or "Harthorne" or "Hawthorne" or "Hedgethorn" or "Maybush" or "Maythorn" or "Mehlbeebaum" or "Meidorn" or "Noble Epine" or "Shen Zha" or "Oneseed Hawthorn" or "Poire d'Oiseaux" or "Sable Epine" or "Shanzha" or "Weissdorn" or "Whitehorn" or "Heliotherapy" or "Heliotrope" or "Hellerwork" or "Deep Tissue Bodywork and Movement Education" or "Structural Integration" or "Helminth Therapy" or "Trichuris suis ova" or "Trichuris trichiura ova" or "Tisane" or "High Fiber Diet" or "Hirudotherapy" or "Hirudin" or "Hirudoterapia" or "Hirudotherapie" or "Leech" or "Leeches" or "Sangsue Anticoagulante" or "Sangsue Medicinale" or "Sangsue Officinale" or "Therapie par les Sangsues" or "Holistic Health" or "Holism" or "Holographic Resonance Repatterning" or "Home Remedies" or "Homeopathy" or "Homeopathic" or "Homeopathie" or "Homeopathique" or "Homeopatia" or "Homeoprophylaxis" or "Maladie Semblable" or "Medecine Homeopathique" or "Nosodes" or "Remede Homeopathique" or "Similar Disease" or "Homoharringtonine" or "Homotoxicology" or "Hoodia" or "Cactus" or "Kalahari Diet" or "Xhoba" or "Humulus lupulus" or "Asperge Sauvage" or "Couleuvree" or "Hopfenzapfen" or "Houblon" or "Lupuli Strobulus" or "Lupulin" or "Lupulo" or "Pi Jiu Hua" or "Salsepareille Indigene" or "Vigne du Nord" or "Aesculus hippocastanum" or "Aescin" or "Buckeye" or "Castano de Indias" or "Chataignier de Mer" or "Chataignier des Chevaux" or "Chestnut" or "Conker Tree" or "Escine" or "Faux-Chataignier" or "Hippocastani Cortex" or "Hippocastani Flos" or "Hippocastani Folium" or "Hippocastani Semen" or "Hippocastanum Vulgare Gaertn" or "Marron Europeen" or "Marronnier" or "Venastat" or "Venostat" or "Venostasin Retard" or "Horticultural Therapy" or "Hoxsey" or "Humor Therapy" or "Huna" or "Huperzine" or "Huperzina A" or "Selagine" or "Hydrazine Sulfate" or "Segidrin" or "Sehydrin" or "Sulfate d'Hydrazine" or "Sulfato de Hidracina" or "Hydrogen Peroxide" or "Immune Boosters" or "Immunoaugmentative Therapy" or "Immuno-Augmentative Therapy" or "Immunoaugmentation" or "Initiatory Medicine" or "Inositol" or "Hexahydroxycyclohexane" or "1,2,3,4,5,6-Cyclohexanehexol, cis-1,2,3,5-trans-4,6-Cyclohexanehexol" or "D-chiro-inositol" or "(+)-chiroinositol" or 1,2,5,3,4,6-inositol or "(1S)-inositol" or "(1S)-1,2,4,3,5,6-inositol" or "Antialopecia Factor" or "Cyclohexito" or "Dambrose" or "D-Myo-Inositol" or "Facteur Anti-Alopecique" or "Inose" or "Inosite" or "Inositol Monophosphate" or "Lipositol" or "Meso-Inositol" or "Monophosphate d'Inositol" or "Mouse Antialopecia Factor" or "Myo-Inositol" or "Vitamin* B8" or "Integrative Medicine" or "Integrated Medicine" or "Integrative Therapy" or "Integrated Therapy" or "Integrative Approach" or "Integrated Approach" or "Integrative Health" or "Integrated Health" or "Interactive Metronome" or "Iodine" or "Atomic number 53" or "Iode" or "Iodide" or "Iodized Salt" or "Iodure" or "Lugol's Solution" or "Numero Atomique 53" or "Periodate de Sodium" or "Potassium Triiodide" or "Sodium Periodate" or "Solution de Lugol" or "Yodo" or "Ipriflavone" or "7-isopropoxyisoflavone" or "7-Isopropoxy Isoflavone" or "Ipriflavona" or "Iridology" or "Bilan Iridologique" or "Diagnostic par l'Iris" or "Irido-Diagnostique" or "Iridiologie" or "Iridologia" or "Iridologist" or "Iridologue" or "Iris" or "Iron" or "Atomic number 26").ti,ab,kf. | 937910 |
| 7 | ("Elemental Iron" or "Ferric Iron" or "Ferric Hydroxide Polymaltose" or "Ferric Orthophosphate" or "Ferric Oxide Saccharide" or "Ferric Sodium Citrate" or "Ferrous Carbonate Anhydrous" or "Ferrous Citrate" or "Ferrous Fumarate" or "Ferrous Gluconate" or "Ferrous Iron" or "Ferrous Pyrophosphate" or "Ferrous Succinate" or "Ferrous Sulfate" or "Ferrum Phosphoricum" or "Hierro" or "Orthophosphate Ferrique" or "Numero Atomique 26" or "Islamic Medicine" or "Isoflavones" or "Jaffe Mellor Techniques" or "Jamu" or "Jasmine" or "Jasminum grandiflorum" or "Jasminum officinale" or "Jasmin" or "Jasmind'Espagne" or "Jati" or "Jazmin" or "Poet's Jessamine" or "Jin Bu Huan" or "Jin Li Da Liquor" or "Jin Shin" or "Kampo Medicine" or "Japanese Medicine" or "Medecine Japonaise" or "Medecine Kampo" or "Medecine Orientale" or "Medecine Traditionnelle Asiatique" or "Medicina Kanpo" or "Medecine Kampo" or "Oriental Medicine" or "Traditional Asian Medicine" or "Kava" or "Piper methysticum" or "Ava Pepper" or "Ava Root" or "Awa" or "Gea" or "Intoxicating Pepper" or "Kao" or "Kavain" or "Kavapipar" or "Kawa" or "Kawapfeffer" or "Kew" or "Lawena" or "Long Pepper" or "Malohu" or "Maluk" or "Meruk" or "Milik" or "Poivre des Cannibales" or "Poivre des Papous" or "Rauschpfeffer" or "Sakau" or "Tonga" or "Waka" or "Wurzelstock" or "Yagona" or "Yangona" or "Yaqona" or "Yaquon" or "Yongona" or "Kneipp Cure" or "Kombucha" or "Algue de The" or "Champagne of Life" or "Champignon de la Charite" or "Champignon des Heros" or "Champignon de Longue Vie" or "Champignon Miracle" or "Combucha Tea" or "Fungus Japonicus" or "Kwassan" or "Manchurian Fungus" or "Mushroom Infusion" or "Petite Mere Japonaise" or "Spumonto" or "T'Chai from the Sea" or "Tschambucco" or "Krebiozen" or "Carcalon" or "Drug X" or "Substance X" or "La'au Lapa'au" or "Laetrile" or "Amygdalin" or "Lam Kam San Heklin" or "2-Amino-5-guanidinopentanoic acid" or "2-Amino-5-(diaminomethylidene amino) pentanoic acid" or "(2S)-2-Amino-5-{[amino (imino) methyl]amino}pentanoic Acid" or "(S)-2-Amino-5- Guanidinopentanoic Acid" or Acide 2-Amino-5-Guanidinopentanoique or "Arginine" or "L-Arginina" or "RGene 10" or "Laser Therapy" or "Laughter Therapy" or "Lavender" or "Lavandula angustifolia" or "Lavandula officinalis" or "Lavandula vera" or "Lavandula spica" or "Lavandula dentata" or "Lavandula latifolia" or "Lavandula pubescens" or "Lavandula stoechas" or "Alhucema" or "Huile Essentielle de Lavande" or "Lavanda" or "Lavande" or "Ostokhoddous" or "Leafflower" or "Phyllanthus" or "Lecithin*" or "Ovolecithin*" or "Phospholipide de Soja" or "Phospholipide* de Soya" or "Soy Phospholipid*" or "Vegilecithin" or "Vitellin*" or "Balm" or "Melissa officinalis" or "Balsamo de Limon" or "Cure-All" or "Dropsy Plant" or "Honey Plant" or "Melisa" or "Melissa" or "Melissae Folium" or "Melisse" or "Melissenblatt" or "Monarde" or "Sweet Mary" or "Toronjil" or "Lentinan" or "Lentinane" or "Lentinus Edodes" or "Polysaccharide" or "Xiangguduotang" or "Xiangguduotangzhusheye" or "Licorice" or "G echinate" or "G glabra" or "G glandulifera" or "G uralensis" or "Acide Glycyrrhizique" or "Acide Glycyrrhizinique" or "Alcacuz" or "Alcazuz" or "Bois Doux" or "Bois Sucre" or "Can Cao" or "Gan Cao" or "Gan Zao" or "Glabra" or "Glycyrrhiza" or "Glycyrrhizae" or "Glycyrrhizic Acid" or "Glycyrrhizinic Acid" or "Isoavone" or "Jethi-Madh" or "Kanzo" or "Lakritze" or "Liquiritiae Radix" or "Liquirizia" or "Liquorice" or "Mulathi" or "Mulethi" or "Orozuz" or "Phytoestrogen" or "Phyto-oestrogene" or "Racine Douce" or "Regalissse" or "Regaliz" or "Reglisse" or "Regliz" or "Subholz" or "Sussholz" or "Sweet Root" or "Yashtimadhu" or "Yashti-Madhu" or "YashtiMadhuka" or "Zhi Gan Cao" or "Balneophototherap*" or "Bath PUVA" or "Dead Sea Climatotherap*" or "Esogetic Colorpuncture" or "Lime" or "Citrus aurantifolia" or "C aurantifolia" or "Citrus medica var acida" or "Citrus acida" or "Citrus limetta var aromatica" or "Limonia aurantifolia" or "Dam's Apple" or "Bara Nimbu" or "Bijapura" or "Citron Vert" or "Citronnier Vert" or "Lima" or "Limette" or "Limetta" or "Limettier" or "Turanj" or "L-Isoleucine" or "Liuwei Dihuang Pills" or "Live Cell Therap*" or "Livingston Wheeler Therap*" or "L-Leucine" or "L-Valine" or "Lymph Therap*" or "Lysine" or "L-2,6-diaminohexanoic acid" or "Hydrochlorure de L-Lysine" or "Lisina" or "L-Lysine" or "L-Lysine HCl" or "L-Lysine Hydrochloride" or "L-Lysine Monohydrochloride" or "Macrobiotic Diet" or "Ma-Pi 2 Diet" or "Macrobiotics" or "Macrobiotism" or "Maggot therap*" or "Biodebridement" or "Biosurg*" or "Biotherap*" or "Larva* therap*" or "Larval Debridement Therap*" or "Maggot Debridement therap*" or "Magnesium" or "Dimagnesium Malate" or "Epsom Salts" or "Magnesia" or "Magnesio" or "Numero Atomique 12" or "Sels d'Epsom" or "Maharishi Amrit Kalish" or "Lalish Method" or "Maintenance therap*" or "G frondose" or "Champignon Dansant" or "Champignon des Fous Dansants" or "Grifola" or "Hen of the Woods" or "Maitake" or "Monkey's Bench" or "Ram's Head" or "Roi des Champignons" or "Sheep's Head" or "Shelf Fungi" or "Manganese" or "Atomic Number 25" or "Manganeso" or "Manganum" or "Sulfate de Manganes" or "Mantras" or "Manual Lymphatic Drainage" or "Marma therap*" or "Marshmallow" or "Althaea officinalis" or "A officinalis" or "Althaea taurinensis").ti,ab,kf. | 489060 |
| 8 | ("Altea" or "Alteia" or "Althaeae Folium" or "Althaeae Radi" or "Althea" or "Althee" or "Guimauve" or "Gulkhairo" or "Herba Malvae" or "Mallards" or "Malvavisco" or "Marsh Maillo" or "Mauve Blanche" or "Mortification Root" or "Sweet Weed" or "Wymote" or "Massage" or "Bindegewebsmassage" or "Petrissage" or "Shiatsu" or "Tui Na" or "Medical Hydrology" or "Medicinal Fungi" or "Medium Chain Triglycerides" or "1,2,3-Propanetriol Trioctanoate" or "Acide Caprique" or "Acide Caprylique" or "Acide Caproique" or "Acide Laurique" or "Capric Acid" or "Caproic Acid" or "Caprylic Acid" or "Caprylic Triglycerides" or "Laurate-Rich MCTs" or "Lauric Acid" or "Medium-Chain Triacylglycerols" or "Medium-Chain Triglycerides" or "Triacylglycerols a Chaine Moyenne" or "Tricaprylin" or "Triglycerides Capryliques" or "Trigliceridos de Cadena Media" or "Trioctanoin" or "Megavitamins" or "Melatonin*" or "N-Acetyl-5-Methoxytryptamine" or "5-Methoxy-N-Acetyltryptamine" or "Pineal Hormone" or "Mental Healing" or "Meridian System" or "Mesotherap*" or "Metamorphic Technique" or "Micronutrients" or "Milk Thistle" or "Silybum marianum" or "S marianum" or "Carduus marianus" or "Artichaut Sauvage" or "Cardo Lechoso" or "Cardui Mariae Fructus" or "Cardui Mariae Herba" or "Carduus Marianum" or "Chardon Argente" or "Chardon de Marie" or "Chardon de Notre-Dame" or "Chardon Marbre" or "Chardon-Marie" or "Epine Blanche" or "Holy Thistle" or "Lady's Thistle" or "Lait de Notre-Dame" or "Legalon" or "Marian Thistle" or "Mariendistel" or "Mary Thistle" or "Shui Fei Ji" or "Silibinin" or "Silybe de Marie" or "Silybin" or "Silybum" or "Silymarin" or "Silymarine" or "St Mary Thistle" or "St Marys Thistle" or "Mind-Body Medicine" or "Mineral Water" or "Mint*" or "Mentha aquatica" or "M aquatica" or "Mentha palustris" or "Baume d'Eau" or "Baume de Riviere" or "Hierbabuena" or "Menta del Agua" or "Menta del Pantano" or "Menta Vellosa" or "Yerbabuena" or "Mentha canadensis" or "M canadensis" or "Mentha arvensis var piperascens" or "Bakha" or "Cornmint" or "Menta Japonesa" or "Mentha Arvensis Aetheroleum" or "Minzol" or "Poleo" or "Pudina" or "Putiha" or "Spearmint" or "Mentha spicata" or "M spicata" or "Mentha viridis" or "Mentha cordifolia" or "Mentha crispa" or "Hierbabuena" or "Menta Verde" or "Pahari Pudina" or "Putiha" or "Sage of Bethlehem" or "Yerba Buena" or "Yerbabuena" or "English Horsemint" or "Mentha longifolia" or "M longifolia" or "Mentha sylvestris" or "Menta de Caballo" or "Pudina" or "Peppermint" or "Mentha x piperita" or "Mentha lavanduliodora" or "Extract of Mentha Piperita" or "Extrait de Mentha Piperita" or "Herba Menthae" or "Huile de Mentha Piperita" or "M Balsamea" or "Menta Pepperita" or "Menta Piperita" or "Mentha Balsamea" or "Mentha Oil" or "Mentha Piperita Extract" or "Mentha Piperita Oil" or "Menthae Piperitae Aetheroleum" or "Menthae Piperitae Folium" or "Menthe" or "Menthol" or "Paparaminta" or "Mistletoe" or "Phoradendron leucarpum" or "P leucarpum" or "Phoradendron avescens" or "Phoradendron serontium" or "Viscum leucarpum" or "Viscum avescens" or "Phoradendron macrophyllum" or "Phoradendron tomentosum" or "Muerdago Americano" or "Viscum album" or "V album" or "All-Heal" or "Banda" or "Blandeau" or "Bois de Sainte-Croix" or "Bouchon" or "Devil's Fuge" or "Drudenfuss" or "Eurixor" or "Guerit-Tout" or "Gui" or "Helixor" or "Herb de Chevre" or "Hexenbesen" or "Hurchu" or "Iscador" or "Isorel" or "Leimmistel" or "Mistlekraut" or "Mistletein" or "Muerdago Europeo" or "Mystyldene" or "Nid de Sorciere" or "Pain de Biques" or "Rini" or "Verquet" or "Vert-Bois" or "Vert de Pommier" or "Visci" or "Vogelmistel" or "Vysorel" or "Molybdenum" or "Atomic number 42" or "Ammonium Molybdate" or "Chelate de Molybdate" or "Chelated Molybdenum" or "Citrate de Molybdene" or "Etrathiomolybdate" or "Molibdeno" or "Molybdate d'Ammonium" or "Molybdate de Sodium" or "Molybdene" or "Sodium Molybdate" or "Morita therap*" or "Movement therap*" or "Moxibustion" or "Acu-Moxi" or "Chinetsukyu (Japanese)" or "Kyutoshin (Japanese)" or "Moxa" or "Okyu (Japanese)" or "Mud therap*" or "Peloid" or "Mudras" or "Mugwort" or "A vulgaris" or "Altamisa" or "Armoise" or "Artemise" or "Artemisia" or "Artemisiae Vulgaris Herba" or "Artemisiae Vulgaris Radix" or "Carline Thistle" or "Felon Herb" or "Gemeiner Beifuss" or "Hierba de San Juan" or "Nagadamni" or "Remise" or "Sailor's Tobacco" or "St. John's Plant" or "Tabac de Saint-Pierre" or "Wild Wormwood" or "Mullein" or "Verbascum densiflorum" or "V densiflorum").ti,ab,kf. | 130357 |
| 9 | ("Verbascum phlomides" or "V phlomides" or "Verbascum thapiforme" or "V thapiforme" or "Verbascum thapsus" or "V thapsus" or "Aaron's Rod" or "Adam's Flannel" or "Beggar's Blanket" or "Blanket Herb" or "Blanket Leaf" or "Bouillon Blanc" or "Bouillon Jaune" or "Candleflower" or "Candlewick" or "Cierge Cotonneux" or "Cierge de Notre-Dame" or "Clot-Bur" or "Clown's Lungwort" or "Cuddy's Lungs" or "Duffle" or "Faux Bouillon-Blanc" or "Feltwort" or "Flannelflower" or "Fleur de Grand Chandelier" or "Fluffweed" or "Gidar Tamaku" or "Gordolobo" or "Hag's Taper" or "Hare's Beard" or "Hedge Taper" or "Higtaper" or "Jacob's Staff" or "Longwort" or "Molene" or "Oreille de Loup" or "Oreille de Saint Cloud" or "Our Lady's Flannel" or "Queue de Loup" or "Rag Paper" or "Shepherd's Club" or "Shepherd's Staff" or "Tabac du Diable" or "Torches" or "Velvet Plant" or "Verbasci Flos" or "Wild Ice Leaf" or "Woolen" or "Myrrh" or "Myrrha" or "Myrrhe" or "Commiphora molmol" or "Commiphora habessinica" or "Commiphora abyssinica" or "Balsamodendrum habessinicum" or "Commiphora madagascariensis" or "Commiphora kataf" or "Commiphora erythraea" or "Amyris kataf" or "Hemprichia erythraea" or "Commiphora species" or "Bdellium" or "Bola" or "Commiphora" or "Didin" or "Didthin" or "Heerabol" or "Mirra" or "Mirrh" or "Mo Yao" or "Murrah" or "Opopanax" or "Resina Commiphorae" or "N-Acetylcysteine" or "Nambudripad's Allergy Elimination therap*" or "Natural Medicine" or "Nature therap*" or "Naturopat*" or "Nettle*" or "Urtica dioica" or "U dioica" or "Urtica urens" or "U urens" or "Bichu" or "Feuille d'Ortie" or "Graine d'Ortie" or "Ortie" or "Ortiga" or "Urtica*" or "Lamium album" or "L album" or "Archangel" or "Archangelique" or "Lamier Blanc" or "Lamii Albi Flos" or "Noni" or "Morinda citrifolia" or "M citrifolia" or "Ba Ji Tian" or "Bois Douleur" or "Canarywood" or "Cheese Fruit" or "Hai Ba Ji" or "Hog Apple" or "Luoling" or "Mengkudu" or "Menkoedoe" or "Mora de la India" or "Morinda" or "Mulberry" or "Mure Indienne" or "Nhau" or "Nono" or "Nonu" or "Pau-Azeitona" or "Rotten Cheese Fruit" or "Ruibarbo Caribe" or "Ura" or "Wild Pine" or "Wu Ning" or "Yor" or "Non-Mainstream Medicine" or "Non-Orthodox Practice" or "Non-Pharmacological Intervention" or "Non-Traditional Medicine" or "Nuad Bo Rarn" or "Nutraceuticals" or "Nutrition therap*" or "Oatmeal Bath" or "Ojeok-San" or "Oleander" or "Nerium indicum" or "Nerium odorum" or "Thevetia peruviana" or "Cascabela thevetia" or "Cerbera thevetia" or "Thevetia neriifolia" or "Adelfa" or "Baladre" or "Exile Tree" or "Huang Hua Jia" or "Jia Zhu Tao" or "Kaner" or "Karvir" or "Karvira" or "Laurel" or "Laurel Rosa" or "Laurier Rose" or "Laurose" or "Lorier Bol" or "Nerier a Feuilles de Laurier" or "Nerion" or "Oleanderblatter" or "Oleandre" or "Oleandri Folium" or "Rose Bay" or "Rose Laurel" or "Soland" or "Olive*" or "Olea europaea" or "O europaea" or "Acide Gras Insature" or "Acide Gras Mono-Insature" or "Acide Gras n-9" or "Acide Gras Omega 9" or "Feuille d'Olivier" or "Huile d'Assaisonnement" or "Huile d'Olive" or "Jaitun" or "Oleae Folium" or "Olivae Oleum" or "Olivo" or "Pulpe d'Olive" or "Salad Oil" or "Sweet Oil" or "M aquifolium" or "Barberry" or "Berberis" or "Mahoni*" or "Mountain-Grape" or "Oregon Grape-Holly" or "Scraperoot" or "Uva de Oregon" or "Vigne de l'Oregon" or "Water-Holly" or "Palo Santo" or "Bursera graveolens" or "B graveolens" or "Panchakarma" or "Pancreatic Extract" or "Passion Flower" or "Passiflora incarnata" or "P incarnata" or "Apricot Vine" or "Burucuya" or "Corona de Cristo" or "Fleischfarbige" or "Fleur de la Passion" or "Fleur de Passiflore" or "Flor de Passion" or "Granadilla" or "Grandilla" or "Grenadille" or "Madre Selva" or "Maracuja" or "Maracuya" or "Maypop" or "Pasionari*" or "Pasiflora" or "Passiflor*" or "Passion Vine" or "Passionaria" or "Passionblume" or "Passionflower" or "Passionsblomma" or "Passionsblumenkraut" or "Water Lemon" or "Passion Fruit" or "Passiflora edulis" or "P edulis" or "Past Life therap*" or "Pastoral Care" or "Pau d'Arco" or "Tabebuia impetiginosa" or "T impetiginosa" or "Handroanthus impetiginosus" or "Tabebuia avellanedae" or "Tabebuia heptaphylla" or "Tabebuia palmeri" or "Tecoma impetiginosa" or "Benier de Guyane" or "Ebene Vert" or "Ipe*" or "Lapacho" or "Lebene" or "Pink Trumpet Tree" or "Quebracho" or "Taheebo" or "Trumpet Bush").ti,ab,kf. | 107267 |
| 10 | ("PC-SPES" or "Pectin*" or "Acide Pectique" or "MCP" or "Pennyroyal" or "Mentha pulegium" or "M pulegium" or "Pulegium vulgare" or "Hedeoma pulegioides" or "Melissa pulegioides" or "Dictame de Virginie" or "Fretillet" or "Lurk-In-The-Ditch" or "Mosquito Plant" or "Piliolerial" or "Poleo" or "Pouliot" or "Pulegium" or "Run-By-The-Ground" or "Squaw Balm" or "Squawmint" or "Stinking Balm" or "Tickweed" or "Pennywort" or "Gotu Kola" or "Centella asiatica" or "C asiatica" or "Hydrocotyle asiatica" or "Brahma-Buti" or "Brahma-Manduki" or "Brahmi" or "Bua-Bok" or "Centella" or "Centellase" or "Divya" or "Hydrocotyle" or "Indischer Wassernabel" or "Idrocotyle" or "Indian Water Navelwort" or "Ji Xue Cao" or "Khulakhudi" or "Luei Gong Gen" or "Luo De Da" or "Madecassol" or "Mandukaparni" or "Manduk Parani" or "Mandukig" or "Marsh Penny" or "Talepetrako" or "Tsubo-kusa" or "Tungchian" or "White Rot" or "Bacopa" or "B monnieri" or "Herpestis monniera" or "Moniera cuneifolia" or "Andri" or "Brahmi" or "Herb of Grace" or "Herpestis Herb" or "Hysope d'Eau" or "Jalanimba" or "Jal-Brahmi" or "Jalnaveri" or "Nira-Brahmi" or "Sambrani Chettu" or "Thyme-Leaved Gratiola" or "Water Hyssop" or "Peony" or "Paeonia lactiflora" or "P lactiflora" or "Paeonia albiflora" or "P albiflora" or "Paeonia mascula" or "P mascula" or "Paeonia arietina" or "P arietina" or "Paeonia caucasica" or "P caucasica" or "Paeonia corallina" or "P corallina" or "Paeonia coriacea" or "P coriacea" or "Paeonia daurica" or "P daurica" or "Paeonia kavachensis" or "P kavachensis" or "Paeonia triternata" or "P triternata" or "Paeonia obovata" or "P obovata" or "Paeonia japonica" or "P japonica" or "Paeonia obovata" or "P obovata" or "Paeonia willmottiae" or "P willmottiae" or "Paeonia officinalis" or "P officinalis" or "Paeonia microcarpa" or "P microcarpa" or "Paeonia paradoxa" or "P paradoxa" or "Paeonia suffruticosa" or "P suffruticosa" or "Paeonia arborea" or "P arborea" or "Paeonia moutan" or "P moutan" or "Paeonia anomala" or "P anomala" or "Paeonia veitchii" or "P veitchii" or "Paeonia beresowskii" or "P beresowskii" or "Paeonia woodwardii" or "P woodwardii" or "Bai Shao" or "Chi Shao" or "Cortex Moutan" or "Jiu Chao Bai Shao" or "Moutan" or "Mu Dan Pi" or "Paeoniae Alba" or "Paeoniae Flos" or "Paeoniae Radix" or "Peonia" or "Piney" or "Pivoine" or "Radix Paeoniae" or "Radix Paeoniae Rubra" or "Shakuyaku" or "Shao Yao" or "Ud Saleeb" or "Udsalam" or "Udsalap" or "Phenylalanine" or "2-amino-3-phenyl-propanoic acid" or "Acide Alpha-aminohydrocinnamique" or "Alpha aminohydrocinnamic Acid" or "Beta-phenyl-alanine" or "Beta-phenyl-alanine" or "D-Phenylalanine" or "DL-Phenylalanine" or "Fenilalanina" or "L-Phenylalanine" or "Phosphatidyl Choline" or "Phosphatidylserine" or "Fosfatidilserina" or "Phosphatidyl Serine" or "PtdSer" or "Soy-PS" or "Phytotherap*" or "Phytomedicine" or "Phytoestrogen" or "Phytoestrol" or "Phytonutrient" or "Phytopharma" or "Phytosterol" or "Plant-Based Medicines" or "Floral therap*" or "Pine-Bark Extract" or "Placebo Plant" or "Plant Extracts" or "Plant Oils" or "Plant-Based Medications" or "Pomegranate" or "Punica granatum" or "P granatum" or "Anardana" or "Dadim" or "Dadima" or "Delima" or "Fruit of the Dead" or "Gangsalan" or "Granaatappel" or "Granad*" or "Granatapfel" or "Grenad*" or "Limoni" or "Melogranato" or "Melograno Granato" or "Pomme Grenade" or "Pomo Granato" or "Pomo Punico" or "PPE" or "Roma" or "Romazeira" or "Romeira" or "Shi Liu Gen Pi" or "Shi Liu Pi" or "Tab Tim" or "Potassium" or "Atomic number 19" or "Numero Atomique 19" or "Potasio" or "Pranic Healing" or "Propolis" or "Acide de Cire d'Abeille" or "Bee Glue" or "Beeswax Acid" or "Cire d'Abeille Synthetique" or "Colle d'Abeille" or "Hive Dross" or "Penicilline Russe" or "Propoleos" or "Russian Penicillin" or "Synthetic Beeswax" or "Yellow Propoli" or "Psyllium" or "Plantago arenaria" or "P arenaria" or "Plantago afra" or "Plantago indica" or "Dietary Fiber" or "Erva-das-Pulgas" or "Fibre Alimentaire" or "Fleaseed" or "Fleawort" or "Flohkraut" or "Flohsamen" or "Herbes aux Puces" or "OEil-de-Chien" or "Pilicaire" or "Plantain" or "Psyllii Semen" or "Psyllion" or "Psyllios" or "Puciere" or "Pucilaire" or "Scharzer Flohsame" or "Zaragatona" or "Plantago ovata" or "P ovata" or "Plantago fastigiata" or "Plantago insularis" or "Plantago ispaghula" or "Plantago decumbens" or "Blond Plantago" or "Che Qian Zi" or "Dietary Fiber" or "Englishman's Foot" or "Fibre Alimentaire").ti,ab,kf. | 354498 |
| 11 | ("Indian Plantago" or "Ipagula" or "Isabgola" or "Isabgul" or "Ispaghul" or "Ispaghula" or "Ispagol" or "Plantaginis Ovatae Semen" or "Plantaginis Ovatae Testa" or "Psilio" or "Psillium Blond" or "Spogel" or "Puerarin" or "Pulsed therap*" or "Pumpkin Seed" or "Purple Sweet Potato" or "Purple Yam" or "Pygeum" or "Prunus africana" or "P africana" or "African Plum Tree" or "African Prune" or "Amande Amere" or "Ciruelo Africano" or "Prunier d'Afrique" or "Pyrrolizidine Alkaloid" or "Qi Gong" or "Ba Duan Jin" or "Biyun Method" or "Chan Mi Gong" or "Chi'I Kung" or "Chi Gung" or "Chi Kung" or "Dantian" or "Trifolium pratense" or "Beebread" or "Clovone" or "Daidzein" or "Genistein" or "Miel des Pres" or "Phytoestrogen" or "Trebol Rojo" or "Trefle Commun" or "Trefle des Pres" or "Trefle Pourpre" or "Trefle Rouge" or "Trefle Rougeatre" or "Trefle Violet" or "Trefoil" or "Trifolium" or "Red Yeast Rice" or "Monascus purpureus" or "Arroz de Levadura Roja" or "Cholestin" or "Hong Qu" or "Hongqu" or "Koji Rouge" or "Mevinolin" or "Monacolin K" or "Monascus" or "Red Koji" or "Red Rice" or "Riz Rouge" or "Rotschimmelreis" or "XueZhiKang" or "Xue Zhi Kang" or "Zhibituo" or "Zhitai" or "Zhi Tai" or "Champignon Basidiomycete" or "Champignon d'Immortalite" or "Ganoderma" or "Ling Chih" or "Ling Zhi" or "Mannentake" or "Mushroom*" or "Reishi" or "Rei-Shi" or "Spirit Plant*" or "Resveratrol*" or "3,4',5-stilbenetriol" or "3,5,4'-trihydroxystilbene" or "3,4',5-trihydroxystilbene" or "3,5,4'-trihydroxy-trans-stilbene" or "3,5,4' TriHydroxy-Transstibene" or "(E)-5-(4-hydroxystyryl)benzene-1,3-diol" or "Cis-Resveratrol" or "Extrait de Vin" or "Kojo-Kon" or "Phytoalexin*" or "Phytoestrogen" or "Phyto-oestrogene" or "Pilule de Vin" or "Protykin" or "Trans-Resveratrol" or "Wine Extract" or "Wine Pill" or "Revici Method" or "Rhodiola" or "Sedum rosea" or "Arctic Root" or "Golden Root" or "Hongjingtian" or "Hong Jing Tian" or "King's Crown" or "Lignum Rhodium" or "Orpin Rose" or "Racine d'Or" or "Racine Doree" or "Rhodiole" or "Rodia Riza" or "Rose Root" or "Rosenroot" or "Roseroot" or "Rosewort" or "Snowdown Rose" or "Rice Bran" or "Oryza sativa" or "Cereal Fiber" or "Dietary Fiber" or "Fibre Alimentaire" or "Fibre Cerealiere" or "Ricebran Oil" or "Riz de Son" or "Salvado de Arroz" or "Son de Riz" or "Safflower Yellow Injection" or "Saffron" or "Crocus sativus" or "Autumn Crocus" or "Azafran" or "Azafron" or "Croci Stigma" or "Crocus Cultive" or "Kashmira" or "Kesar" or "Kumkuma" or "Safran" or "Zafran" or "Salacia" or "Chundan" or "Kothala Himbutu" or "Ponkoranti" or "S oblonga" or "Diviners Sage" or "Divinorin" or "Feuilles de la Bergere" or "Feuilles de la Vierge" or "Herb-of-the-Virgin" or "Herb of Mary" or "Herba de Maria" or "Hierba de la Virgen" or "Hierba Maria" or "Hojas de la Pastora" or "Hojas de Maria" or "La Hembra" or "Leaf of Mary" or "Menthe Magique" or "Mexican Sage" or "Pipiltzintzintli" or "Sadi" or "Sally-D" or "Salvia" or "Salvinorin" or "Sage of the Seers" or "Sauge des Devins" or "Sauge Divinatoire" or "Shepherdess" or "Ska Maria" or "Ska Pastora" or "Yerba de Maria" or "Yerba Maria" or "Samadhi" or "S-adenosyl-L-methionine" or "Ademetionine" or "Adenosylmethionine" or "S-Adenosyl Methionine" or "S-Adenosyl-L-Methionine" or "S-Adenosylmethionine" or "SAM-e" or "Samyr" or "Samyama" or "Sanchi Preparation" or "Sand Bath" or "Sauna" or "Saw Palmetto" or "Serenoa repens" or "S repens" or "Serenoa serrulata" or "S serrulata" or "Sabal serrulata" or "American Dwarf Palm Tree" or "Baies du Palmier Scie" or "Cabbage Palm" or "Chou Palmiste" or "Ju-Zhong" or "Palma Enana Americana" or "Palmier de Floride" or "Palmier Nain" or "Palmier Scie" or "Sabal" or "Saw Palmetto Berry" or "Scraping" or "Coining" or "Gua Sha" or "Kerokan Spooning" or "Seaweed" or "Sectarian Medicine" or "Selenium" or "Atomic number 34" or "Ebselen" or "L-Selenomethionine" or "Levure Selenisee" or "Numero Atomique 34" or "Selenio" or "Selenite" or "Selenized Yeast" or "Selenomethionine" or "Senna" or "Casse" or "Fan Xie Ye" or "Sen" or "Sena Alejandrina" or "Sene" or "Sennae Folium" or "Sennae Fructus" or "Sennosides" or "Sensory Therap*" or "Shamanism" or "Shamanic Healing" or "Shengmai" or "Shenmai" or "Shenqi Fuzheng" or "Shensu" or "Shenfu" or "Shexiang" or "Shiatsu" or "Finger Pressure" or "Medecine Energetique" or "Ohashiatsu" or "Sante Energetique" or "Shi-astsu" or "Shiatsupractor" or "Lentinus edodes" or "Lenticus edodes" or "Lentinula edodes" or "Tricholomopsis edodes" or "Champignon Noir" or "Champignon Parfume" or "Hua Gu" or "Lentin" or "Lentinula" or "Pasania Fungus" or "Shiitake" or "Shitake" or "Snake Butter" or "Xiang-Gu" or "Shuanghuanglian" or "Siddha" or "Silicon" or "Si" or "Atomic number 14" or "Acide Orthosilicique" or "Numero Atomique 14" or "Orthosilicic Acid" or "Polysilicone-11" or "Silica" or "Silice Hydride" or "Silicea" or "Silicio" or "Silicium" or "Sodium Silicate" or "Sintergetic Medicine" or "Snake Venom" or "Soy" or "Glycine max" or "Glycine gracilis" or "G gracilis" or "Glycine hispida" or "Phaseolus max" or "Daidzein*" or "Edamame" or "Estrogene Vegetal" or "Genistein*" or "Isoflavone*" or "Legume" or "Miso" or "Natto" or "Phytoestrogen" or "Phyto-oestrogene" or "Plant Estrogen" or "Shoyu" or "Soja" or "Sojabohne" or "Soya" or "Soybean" or "Tempeh" or "Texturized Vegetable Protein" or "Tofu" or "Touchi" or "St John's Wort" or "Hypericum perforatum" or "H perforatum" or "Amber" or "Barbe de Saint-Jean" or "Chasse-Diable" or "Demon Chaser" or "Fuga Daemonum" or "Goatweed" or "Hardhay" or "Hierba de San Juan" or "Hypereikon" or "Hyperici Herba" or "Hypericum" or "Klamathaweed" or "Millepertuis" or "Rosin Rose" or "Saynt Johannes Wort" or "Stanol therap*" or "Sitostanol" or "3-beta,5-alpha-stigmastan-3-ol" or "24-alpha-ethylcholestanol" or "Beta-sitostanol" or "Dihydro-beta-sitosterol" or "Fucostanol" or "Phytostanol" or "Plant Stanol" or "Stanol Vegetal" or "Stigmastanol" or "Plant Sterol" or "(24S)-5,22-Stigmastadien-3-beta-ol" or "3-beta-stigmast-5-en-3-ol, 22,23-dihydrostigmasterol" or "24-beta-ethyl-delta-5-cholesten-3-beta-ol" or "24-ethyl-cholestero" or "Avenasterol" or "B-sitosterol 3-B-D-glucoside" or "B-Sitosterolin" or "B-Sitosterols" or "Beta-sitosterine" or "Beta-Sitosterol" or "Betasitosterol" or "Brassicasterol" or "Campest-5-en-3beta-ol" or "Campesterol" or "Cinchol" or "Cupreol" or "Dihydro-beta-sitosterol" or "Ester de Sterol Vegetal" or "Esters de Sterol Derives d'huile Vegetale" or "Phytosterol*" or "Plant Sterolins" or "Quebrachol" or "Rhamnol" or "Sitosterin" or "Sitosterol*" or "Sterinol" or "Sterolin*" or "Sterols Vegetaux" or "Stigmasterin" or "Stigmasterol" or "Vegetable Oil Sterol Esters" or "Vegetable Sterol Esters" or "Staphage Lysate" or "Stevia" or "Eupatorium rebaudianum" or "Mustelia eupatoria" or "Azucacaa" or "Caa-He-E" or "Caa'Inhem" or "Ca-A-Jhei" or "Ca-A-Yupi" or "Capim Doce" or "Chanvre d'Eau" or "Eira-Caa" or "Erva Doce" or "Estevia" or "Kaa Jhee" or "Plante Sucree" or "Reb A" or "Rebaudioside A" or "Rebiana" or "Stevioside" or "Sweet Herb" or "Sweet Leaf of Paraguay" or "Sweetleaf" or "Yerba Dulce" or "Tao" or "Taurine" or "2-Aminoethane Sulfonic Acid" or "2-Aminoethylsulfonic Acid" or "Aminoethanesulfonate" or "Aminoethylsulfonique" or "Dibicor" or "L-Taurine" or "Taurina" or "Taurine Ketoisocaproic Acid" or "Melaleuca alternifolia" or "M alternifolia" or "Aceite del Arbol de Te" or "Huile de Melaleuca" or "Huile de Theier" or "Huile Essentielle de Theier" or "Melaleuca Oil" or "Oil of Melaleuca" or "Oleum Melaleucae" or "Tea Tree" or "Ti Tree Oil" or "Threonine" or "L-Threonine" or "Treonina" or "Thunder God Vine" or "Tripterygium wilfordii" or "Huang-T'eng Ken" or "Lei Gong Teng" or "Lei-Kung T'eng" or "Seven Step Vine" or "Taso-Ho-Hua" or "Threewingnut" or "Tonnerre de la Vigne de Dieu" or "Tripterigium Wilfordii" or "Vigne du Tonnerre Divin" or "Yellow Vine" or "Thyme" or "Farigoule").ti,ab,kf. |  |
| 12 | ("Farigoulette" or "Frigoule" or "Mignotise des Genevois" or "Pote" or "Serpolet" or "Thym" or "Thymi herba" or "Tomillo" or "Van Ajwayan" or "Vanya Yavani" or "Iper" or "Serpyllum" or "Complexe de Peptides Thymiques" or "Extracto de Timo" or "Extrait Thymique" or "Proteine Thymique" or "Thymic Extract" or "Thymic Peptide" or "Thymic Protein" or "Thymomodulin" or "Thymosin*" or "Thymostimulin" or "Thymostimuline" or "Thymus" or "Thymus-Derived Polypeptides" or "Tianmadingxian Capsule" or "Tinospora cordifolia" or "Ambervel" or "Amrita" or "Amritha" or "Gilo" or "Giloe" or "Giloy" or "Glunchanb" or "Guduchi" or "Gulancha Tinospora" or "Gulvel" or "Gurcha" or "Heavenly Elixir" or "Indian Tinospora" or "Jetwatika" or "Mehahara" or "Mehaghna" or "Moonseed" or "Pramehahara" or "Pramehaghna" or "Sindal" or "Sittamrytu" or "Somida" or "Tinospora" or "Tinosporia Cordifolus" or "Tissue Therap*" or "Topical Therap*" or "Turmeric" or "Curcuma" or "Curcumae Longa" or "Curcumae Longae Rhizoma" or "Curcumin*" or "Halada" or "Haldi" or "Haridra" or "Indian Saffron" or "Nisha" or "Pian Jiang Huang" or "Radix Curcumae" or "Rajani" or "Rhizoma Cucurmae Longae" or "Yu Jin" or "Curcumae Xanthorrhizae Rhizoma" or "Temoe-Lawacq" or "Temoe-Lawaq" or "Temu Lawak" or "Temu Lawas" or "Tewon Lawa" or "B aristata" or "Chitra" or "Citra" or "Darhahed" or "Darhald" or "Daruhaldi" or "Daruharidra" or "Darurajani" or "Darvi" or "Epine-Vinette Aristee" or "Hint Amberparisi" or "Indian Berberry" or "Indian Lycium" or "Pisse Vinaigre" or "Vinettier Ariste" or "Tyrosine" or "2-Acetylamino-3-(4-Hydroxyphenyl)-Propanoic Acid" or "2-Amino-3-(4-Hydroxyphenyl)-propionic acid" or "Acetyl-L-Tyrosine" or "L-Tyrosine" or "N-Acetyl-L-Tyrosine" or "N-Acetyl-Tyrosine" or "Tirosina" or "Tyrosinum" or "Graeco-Arabic Medicine" or "Medecine Greco-Arabe" or "Unaani" or "Unani" or "Yunani" or "Mutra Paribhasa" or "Mutra Varga" or "Naramutra" or "Shivambu" or "Uro-therap*" or "Urotherap*" or "Valerian" or "All-Heal" or "Amantilla" or "Baldrian" or "Baldrianwurzel" or "Garden Heliotrope" or "Guerit Tout" or "Tagar" or "Tagar-Ganthoda" or "Tagara" or "Valeriana" or "Valerianae Radix" or "Valeriane" or "Centranthus ruber" or "Alfenique" or "Barbe de Jupiter" or "Bouncing Bess" or "Bovis and Soldier" or "Centranthe Rouge" or "Centranto" or "Delicate Bess" or "Drunken Sailor" or "Fox's-Brush" or "Jupiter's Beard" or "Lilas d'Espagne" or "Milamores" or "Pretty Betsy" or "Vanadium" or "Atomic number 23" or "Metavanadate" or "Orthovanadate" or "Vanadate" or "Vanadio" or "Vanadyl" or "Vanilla" or "Myrobroma fragrans" or "Vainilla" or "Vanille" or "Vitamin A" or "3-Dehydroretinol" or "Antixerophthalmic Vitamin" or "Axerophtholum" or "Dehydroretinol" or "Fat-Soluble Vitamin" or "Oleovitamin A" or "Retinoid*" or "Retinol" or "Retinyl Acetate" or "Retinyl Palmitate" or "Retinyl Palmitate" or "Vitamin A1" or "Vitamin A2" or "Vitamina A" or "Vitamine A" or "Vitamine A1" or "Vitamine A2" or "Vitamine Liposoluble" or "Vitaminum A" or "Vitamin B12" or "Cyanocobalamin*" or "Hydroxocobalamin*" or "Methylcobalamin*" or "B-12" or "B12" or "B Complex" or "Bedumil" or "Cobalamin*" or "Cobamin*" or "Complexe Vitaminique B" or "Cycobemin" or "Hydroxocobemine" or "Idrossocobalamina" or "Vitadurin*" or "Vitamina B12" or "Vitamine B12" or "Vitamin B2" or "Riboflavin*" or "Complexe de Vitamines B" or "Flavin*" or "Lactoflavin*" or "Vitamin G" or "Vitamina B2" or "Vitamine B2" or "Vitamine G" or "Vitamin B3" or "Niacin*" or "3-Pyridinecarboxylic Acid" or "Nicotinic acid" or "Acide Nicotinique" or "Acide Pyridine-Carboxylique-3" or "Anti-Blacktongue Factor" or "Antipellagra Factor" or "Facteur Anti-Pellagre" or "Nicosedine" or "Pellagra Preventing Factor" or "Vitamin PP" or "Vitamina B3" or "Vitamine B3" or "Vitamine PP" or "Niacinamide" or "Pyridine-3-carboxamide" or "3-Pyridine Carboxamide" or "3-Pyridinecarboxamide" or "Amide de l'Acide Nicotinique" or "Niacinamida" or "Nicamid" or "Nicosedine" or "Nicotinamide" or "Nicotinic Acid Amide" or "Nicotylamidum" or "Vitamin B3a" or "Vitamina B3" or "Vitamine B3" or "Vitamin B5" or "D-Pantothenic Acid" or "Pantothenic Acid" or "Acide D-Pantothenique" or "Acide Pantothenique" or "Acido Pantotenico" or "Alcool Pantothenylique" or "Calcii P antothenas" or "D-Panthenol" or "D-Pantothenate de Calcium" or "D-Pantothenyl Alcohol" or "Dexpanthenol" or "Dexpanthenolum" or "Pantethine" or "Panthenol" or "Pantothenate" or "Pantothenate" or "Pantothenol" or "Pantothenylol" or "Vitamin B-5" or "Vitamina B5" or "Vitamine B5" or "Vitamin B6" or "Pyridoxine" or "Pyridoxal" or "Pyridoxamine" or "Pyridoxine-5'-Phosphate" or "Pyridoxal-5'-Phosphate" or "Pyridoxamine-5'-Phosphate" or "Adermine Chlorhydrate" or "Adermine Hydrochloride" or "B6" or "Phosphate de Pyridoxal" or "Piridoxina" or "Pyridoxal Phosphate" or "Pyridoxal 5 Phosphate" or "Pyridoxal-5-Phosphate" or "Pyridoxine-5-Phosphate" or "P5P" or "P-5-P" or "Vitamin B-6" or "Vitamina B6" or "Vitamine B6" or "Vitamin B7" or "Biotin" or "Biotina" or "Biotine" or "Biotine-D" or "Coenzyme R" or "D-Biotin" or "Vitamin H" or "Vitamine B7" or "Vitamine H" or "W Factor" or "Vitamin C" or "Ascorbic Acid" or "Acide Ascorbique" or "Acide Cevitamique" or "Acide Iso-Ascorbique" or "Acide L-Ascorbique" or "Acido Ascorbico" or "Antiscorbutic Vitamin" or "Ascorbate" or "Ascorbyl Palmitate" or "Cevitamic Acid" or "Iso-Ascorbic Acid" or "L-Ascorbic Acid" or "Palmitate d'Ascorbyl" or "Vitamina C" or "Vitamine Antiscorbutique" or "Vitamine C" or "Vitamin D" or "1, 25-Dihydroxycholecalciferol" or "25-Hydroxycholecalciferol" or "Alfacalcidol" or "Calcifediol" or "Calcipotriene" or "Calcitriol" or "Cholecalciferol" or "Dihydrotachysterol" or "Ergocalciferol" or "Paricalcitol" or "Alfacalcidol" or "1-Alpha-Hydroxycholecalciferol" or "1-Alpha-Hydroxycholecalciferol" or "1alpha(OH)D3" or "Vitamina D" or "Vitamine D" or "25-HCC" or "25-hydroxycholecalciferol" or "25-hydroxyvitamin D3" or "25-hydroxyvitamine D3" or "25-OHCC" or "25-OHD3" or "Calcipotriene" or "Calcipotriol" or "1,25-DHCC" or "1,25-dihydroxycholecalciferol" or "1,25-dihydroxyvitamin D3" or "1,25-dihydroxyvitamine D3" or "1,25-diOHC" or "1,25(OH)2D3" or "Cholecalciferol" or "7-dehydrocholesterol Active" or "Activated 7-dehydrocholesterol" or "Colecalciferol" or "Vitamin D3" or "Dihydrotachysterol" or "dihydrotachysterol 2" or "dichysterol" or "Vitamine D3" or "Ergocalciferol" or "Activated Ergosterol" or "Calciferol" or "Ergocalciferol" or "Ergocalciferolum" or "Ergosterol Active" or "Irradiated Ergosterol" or "Ergosterol Irradie" or "Viosterol" or "Vitamin D2" or "Vitamine D2" or "Paricalcitol" or "19-nor-1,25-dihydroxyvitamin D2" or "19-nor-1,25-dihydroxyvitamine D2" or "Paracalcin" or "Vitamin E" or "Alpha-Tocopherol" or "Beta-Tocopherol" or "Delta-Tocopherol" or "Gamma-Tocopherol" or "Acetate d'Alpha Tocopheryl" or "Acetate de Tocopherol" or "Acetate de Tocopheryl" or "All-Rac-Alpha-Tocopherol" or "Beta-Tocotrienol" or "D-Alpha-Tocopherol" or "D-Alpha-Tocopheryl" or "DL-Alpha-Tocopherol" or "DL-Alpha-Tocopheryl" or "D-Tocopherol" or "DL-Tocopherol" or "D-Beta-Tocopherol" or "D-Delta-Tocopherol" or "Delta-Tocotrienol" or "Delta-Tocopherol" or "D-Gamma-Tocopherol" or "D-Gamma-Tocotrienol" or "DL-Tocopherol" or "Gamma-Tocotrienol" or "RRR-Alpha-Tocopherol" or "Succinate Acide de Tocopheryl" or "Tocopherol*" or "Tocotrienol*" or "Vitamina E" or "Vitamine E" or "Vitamine Liposoluble" or "Vitamin K" or "Phytonadione" or "Menaquinone" or "Menadione" or "Menadiol" or "4-amino-2-methyl-1-naphthol" or "Vitamin K1" or "Methylphytyl Naphthoquinone" or "Phylloquinone" or "Phytomenadione" or "Phytonadione" or "2-Methyl-3-Phytyl-1,4-Naphthoquinone" or "Vitamina K" or "Vitamine K" or "Vitamine Liposoluble" or "Vitamine Soluble dans les Graisses" or "Vitamin K2" or "Menatetrenone" or "Vitamin K3" or "2-Methyl-1,4-Naphthoquinone" or "Vitamin K4" or "Menadiolum Solubile Methylnaphthohydroquinone" or "Vitamin K5" or "Vitamins" or "Agneau du Moine" or "Agneau-chaste" or "Agni Casti" or "Agnocasto" or "Agnolyt" or "Agnus-Castus" or "Chaste Berry" or "Chaste Tree" or "Chasteberry" or "Chastetree" or "Gattilier" or "Hemp Tree" or "Mang Jing Zi" or "Panj-Angosht" or "Pimiento del Monje" or "Vitex" or "Viticis Fructus" or "Vojta Method" or "Western Herbal Medicine" or "Native American Herbs" or "Western Herbalism" or "Western Herbs" or "White Willow" or "Salix alba" or "European Willow" or "Wigmore Diet" or "Wild Medicine" or "Avellano de Bruja" or "Cafe du Diable" or "Hamamelis" or "Hazel" or "Noisetier des Sorcieres" or "Snapping Tobacco Wood" or "Spotted Elder" or "Winter Bloom" or "Xiaxingci Granule" or "Yarrow" or "Achilee" or "Achillee" or "Achillea" or "Acuilee" or "Band Man's Plaything" or "Bauchweh" or "Birangasifa" or "Birangasipha" or "Biranjasipha" or "Bloodwort" or "Bumadaran" or "Civan Percemi" or "Devil's Nettle" or "Devil's Plaything" or "Erba Da Cartentieri" or "Erba Da Falegname" or "Gandana" or "Gemeine Schafgarbe" or "Green Arrow" or "Katzenkrat" or "Little Feather" or "Milefolio" or "Milenrama" or "Milfoil" or "Millefeuille" or "Millefolii Flos" or "Millefolii Herba" or "Millefolium" or "Millegoglie" or "Plumajillo" or "Rajmari" or "Roga Mari" or "Sanguinary" or "Sourcil de Venus" or "Staunchweed" or "Tausendaugbram" or "Thousand-Leaf" or "Wound Wort" or "Yohimbe" or "Corynanthe Johimbe" or "Johimbi" or "Yohimbehe" or "Yohimbine" or "Yohimbinum Muriaticum" or "Zhixian I Pill" or "Zinc" or "Zn" or "Atomic number 30" or "Numero Atomique 30" or "Polaprezinc" or "Zincum Aceticum" or "Zincum Gluconicum" or "Zincum Metallicum" or "Zincum Valerianicum" or "Zishen Tongli Jianonang").ti,ab,kf. | 1048746 |
| 13 | herbal medicine/ or exp plant extracts/ or exp Phytotherapy/ or exp Plant Oils/ or exp medicine, traditional/ or exp complementary therapies/ or exp Cannabinoids/ or (((herb* or plant* or botanical or phyto* or tradition* or indigenous or folk or natural or holistic or unconventional or alternative or Persian or African or Arabic or Ayurvedic or East Asian or Chinese or Kampo or Korean or Mongolian or Tibetan or Iranian or Thai) adj3 (medicin* or remed* or heal*)) or Shamanism or complementary therap*).ti,ab,kf. | 667898 |
| 14 | Peripheral Nervous System Diseases/ci or (chemotherapy-induced peripheral neuropathy or CIPN).ti,ab,kf. | 6377 |
| 15 | exp administration, topical/ or (topical* or powder* or oil* or lotion* or spray* or tincture* or gel* or foam* or cream* or ointment* or paste* or powder* or essential or external).ti,ab,kf. | 2481340 |
| 16 | 1 or 2 or 3 or 4 or 5 or 6 or 7 or 8 or 9 or 10 or 11 or 12 or 13 | 5543905 |
| 17 | 14 and 15 and 16 | 111 |

2. Scopus (ran this search, and then a combination of the first two lines (CIPN and topical treatments) with the longer list of treatment names in the Ovid Medline search)

(TITLE-ABS-KEY ( ( "Peripheral Nervous System Diseases" OR "chemotherapy-induced peripheral neuropathy" OR CIPN ) AND ( topical OR powder OR oil OR lotion OR spray OR tincture OR gel OR foam OR cream OR ointment OR paste OR powder OR external ) )) AND (TITLE-ABS-KEY ( ( Herb OR plant OR phyto* OR ( plant W/2 ( oil* OR extract* ) ) OR "complementary therap*" OR cannabi* OR ( ( botanic* OR tradition* OR indigenous OR folk OR natural OR holistic OR unconventional OR alternative OR Persian OR African OR Arabic OR Ayurved* OR "East Asian" OR Chinese OR Kampo OR Korean OR Mongolian OR Tibetan OR Iranian OR Thai ) W/3 ( medicin* OR remed* OR heal* ) ) OR shamanism ) ))

3. CINAHL (ran only abbreviated search due to issues in EBSCO platform)

MH "Medicine, Herbal+" OR MH "Plant Extracts+" OR MH "Plant Oils+" OR MH "Medicine, Traditional+" OR MH "Alternative Therapies+" OR MH "Cannabinoids+" OR XB (((herb* or plant* or botanical or phyto* or tradition* or indigenous or folk or natural or holistic or unconventional or alternative or Persian or African or Arabic or Ayurvedic or East Asian or Chinese or Kampo or Korean or Mongolian or Tibetan or Iranian or Thai) N3 (medicin* or remed* or heal*)) or Shamanism or complementary therap*)

AND

MH "Peripheral Nervous System Diseases/CI" OR XB (chemotherapy-induced peripheral neuropathy or CIPN)

AND

MH "Administration, Topical+" or XB (topical* or powder* or oil* or lotion* or spray* or tincture* or gel* or foam* or cream* or ointment* or paste* or powder* or essential or external)

4. Cochrane Central (ran only this abbreviated search due to platform issues)

ID	Search	Hits

#1	MeSH descriptor: [Herbal Medicine] explode all trees	96

#2	MeSH descriptor: [Plant Extracts] explode all trees	11112

#3	MeSH descriptor: [Phytotherapy] explode all trees	4998

#4	MeSH descriptor: [Plant Oils] explode all trees	2811

#5	MeSH descriptor: [Medicine, Traditional] explode all trees	2334

#6	MeSH descriptor: [Complementary Therapies] explode all trees	30144

#7	MeSH descriptor: [Cannabinoids] explode all trees	1608

#8	(((herb* or plant* or botanical or phyto* or tradition* or indigenous or folk or natural or holistic or unconventional or alternative or Persian or African or Arabic or Ayurvedic or "East Asian" or Chinese or Kampo or Korean or Mongolian or Tibetan or Iranian or Thai) NEAR/3 (medicin* or remed* or heal*)) or Shamanism or (complementary NEXT therap*)):ti,ab,kw (Word variations have been searched)	37604

#9	#1 OR #2 OR #3 OR #4 OR #5 OR #6 OR #7 OR #8	71653

#10	MeSH descriptor: [Peripheral Nervous System Diseases] explode all trees and with qualifier(s): [chemically induced - CI]	476

#11	(("chemotherapy-induced peripheral neuropathy" or CIPN)):ti,ab,kw (Word variations have been searched)	920

#12	#10 OR #11	1223

#13	MeSH descriptor: [Administration, Topical] explode all trees	18830

#14	((topical* or powder* or oil* or lotion* or spray* or tincture* or gel* or foam* or cream* or ointment* or paste* or powder* or essential or external)):ti,ab,kw (Word variations have been searched)	175052

#15	#13 OR #14	181939

#16	#9 AND #12 AND #15	15
